# AII amacrine cells: quantitative reconstruction and morphometric analysis of electrophysiologically identified cells in live rat retinal slices imaged with multi-photon excitation microscopy

**DOI:** 10.1007/s00429-016-1206-0

**Published:** 2016-03-07

**Authors:** Bas-Jan Zandt, Jian Hao Liu, Margaret Lin Veruki, Espen Hartveit

**Affiliations:** Department of Biomedicine, University of Bergen, Jonas Lies vei 91, 5009 Bergen, Norway

**Keywords:** Retina, Rod pathway, Dendrites, Morphology, Morphometry, Branching pattern

## Abstract

AII amacrine cells have been found in all mammalian retinas examined and play an important role for visual processing under both scotopic and photopic conditions. Whereas ultrastructural investigations have provided a detailed understanding of synaptic connectivity, there is little information available with respect to quantitative properties and variation of cellular morphology. Here, we performed whole-cell recordings from AII amacrine cells in rat retinal slices and filled the cells with fluorescent dyes. Multi-photon excitation microscopy was used to acquire image stacks and after deconvolution, we performed quantitative morphological reconstruction by computer-aided manual tracing. We reconstructed and performed morphometric analysis on 43 AII amacrine cells, with a focus on branching pattern, dendritic lengths and diameters, surface area, and number and distribution of dendritic varicosities. Compared to previous descriptions, the most surprising result was the considerable extent of branching, with the maximum branch order ranging from approximately 10–40. We found that AII amacrine cells conform to a recently described general structural design principle for neural arbors, where arbor density decreases proportionally to increasing territory size. We confirmed and quantified the bi-stratified morphology of AII amacrine cells by analyzing the arborizations as a function of retinal localization or with Sholl spheres. Principal component and cluster analysis revealed no evidence for morphological subtypes of AII amacrines. These results establish a database of morphometric properties important for studies of development, regeneration, degeneration, and disease processes, as well as a workflow compatible with compartmental modeling.

## Introduction

Neurons are the main cellular components of the networks in the central nervous system that give rise to a rich variety of neural functions. Common to all neurons is the presence of multiple branching processes with specialized morphology, and neurons in different regions of the central nervous system display an enormous degree of variability, especially in their dendritic morphology (Cajal 1909, 1911). The morphology of a neuron can have a dramatic impact on its function (Mainen and Sejnowski 1996). In general, the computational and signal processing properties of a neuron are determined by its synaptic inputs, its three-dimensional (3D) dendritic morphology and the properties and location of the different ion channels expressed in the cell membrane. Whereas the strongest focus traditionally has been on the morphological variability between different types of neurons, the inherent variability in dendritic morphology within cells belonging to the same neuronal type is often overlooked (Schneider et al. 2014).

The focus in the present study is on the AII amacrine cell, traditionally considered an axon-less interneuron, which is found in all mammalian retinas investigated and plays an important role in both scotopic and photopic processing of visual signals (for reviews, see Demb and Singer 2012; Hartveit and Veruki 2012). The AII amacrine cell was first explicitly characterized in cat retina by Kolb and Famiglietti (1974), but was most likely observed already by Cajal (1892, 1894, 1911). Its existence as a unique type of neuron is based on a characteristic morphology (Kolb and Famiglietti 1974; Famiglietti and Kolb 1975) and an arrangement in a regular mosaic across the retina (Vaney 1985; Mills and Massey 1991; Wässle et al. 1993; Casini et al. 1995). There is a surprising lack of information, however, with respect to the quantitative aspects of AII amacrine cell morphology, including information about the variability of morphological properties. Such information will be important for understanding how the specific morphology of AII amacrine cells impacts their computational properties.

Reconstructions of AII amacrine morphology based on ultrastructural imaging continue to provide new and important information about synaptic connectivity (Tsukamoto and Omi 2013; Marc et al. 2014), but it is currently not feasible to use this approach for a larger population analysis. With light microscopic imaging, there are several different alternatives available for quantitative morphological reconstruction and analysis of single neurons. The Golgi method, employed in both classical and modern studies of neuronal morphology, suffers from the drawback that while it can provide complete morphological visualization at high resolution, it cannot be extended to a workflow that also encompasses correlated measurements of physiological properties from the same neurons. Filling neurons with fluorescent dyes via injection with sharp microelectrodes in fixed tissue slices can also yield excellent morphology (e.g. Dumitriu et al. 2011), but suffers from the same drawback as Golgi impregnation with respect to the inability of obtaining correlated physiological measurements. Cells can also be filled in live tissue (in vitro or in vivo) with tracers such as biocytin (Horikawa and Armstrong 1988) and Neurobiotin (Kita and Armstrong 1991) or with fluorescent dyes, using either sharp microelectrodes or patch pipettes. Importantly, these techniques offer the opportunity of correlated morphological and physiological investigations (Jaeger 2001; Blackman et al. 2014). The use of tracers, however, requires post-processing with tissue fixation before the filled cells can be visualized, and is therefore often accompanied by variable tissue shrinkage which can compromise and distort exact morphological reconstruction (Jaeger 2001). Imaging dye-filled neurons by wide-field fluorescence microscopy suffers from the lack of optical sectioning and is not adequate for detailed morphological reconstruction. Confocal laser-scanning microscopy provides optical sectioning and high resolution, but is difficult to employ for imaging complete neuronal morphologies from live tissue because of problems with phototoxicity (Murphy and Davidson 2013). Confocal microscopy is well-suited for imaging dye-filled neurons after tissue fixation, but that again introduces potential problems with tissue shrinkage and distortion.

Multi-photon excitation (MPE) microscopy is a relatively new technique (Denk et al. 1990) that combines the advantages of several earlier approaches and has few limitations and disadvantages. The resolution is almost as high as confocal microscopy and because of low phototoxicity MPE microscopy is well suited for imaging live tissue in combination with physiological measurements (Tashiro et al. 2006; Groh and Krieger 2011). Imaging in live tissue also eliminates the need to post-process tissue, thus avoiding artifacts related to shrinkage caused by fixation and compression by glass cover slips. A major goal of the present study was to take advantage of the unique opportunities offered by MPE microscopy for high-resolution 3D imaging of live tissue. This allows for a detailed and quantitative morphological analysis based on accurate, digital reconstruction of single neuron morphology and the establishment of a database of quantitative population data that can serve as essential building blocks for anatomically realistic retinal network models (e.g. Gleeson et al. 2007). Such information is essential for understanding the structure–function relationship for any type of neuron.

## Materials and methods

### Retinal slice preparation

General aspects of the methods have previously been described in detail (Hartveit 1996). Albino rats (female; 4–7 weeks postnatal) were deeply anaesthetized with isoflurane in oxygen and killed by cervical dislocation (procedure approved under the surveillance of the Norwegian Animal Research Authority). Anesthesia, dissection, and preparation of slices were done under normal room illumination. During recording, the room lights were dimmed and the experimental setup was screened from room and monitor lights by black cloth. After dissecting the retina from the eyecup, each retina was cut into four quadrants that were stored in an interface chamber with Ames’ solution continuously bubbled with 95 % O_2_–5 % CO_2_ (pH 7.4). Each quadrant was used to prepare a set of vertical retinal slices that were cut by hand with a curved scalpel blade at a thickness of 150–200 µm. A single set of slices was used for 3–4 h before it was replaced by a new set. The slices were visualized using a custom-modified “Movable Objective Microscope” (MOM; Sutter Instrument, Novato, CA, USA) with a 20× water immersion objective (XLUMPLFL; 0.95 NA; Olympus) and infrared Dodt gradient contrast (IR-DGC) videomicroscopy (Luigs & Neumann, Ratingen, Germany) (Dodt et al. 1998), using an IR-sensitive analog CCD camera (VX55; TILL Photonics, Gräfelfing, Germany). Electrophysiological recording and imaging were carried out at room temperature (22–25 °C).

### Solutions and electrophysiological recording

The extracellular perfusing solution was continuously bubbled with 95 % O_2_–5 % CO_2_ and had the following composition (in mM): 125 NaCl, 25 NaHCO_3_, 2.5 KCl, 2.5 CaCl_2_, 1 MgCl_2_, 10 glucose, pH 7.4. The recording pipettes were filled with an intracellular solution of the following composition (in mM): 125 potassium gluconate, 5 KCl, 8 NaCl, 0.2 EGTA, 10 Hepes, 4 MgATP and 0.3 Na_3_GTP (pH was adjusted to 7.3 with KOH). The pipette solution also contained either Alexa Fluor 488 hydrazide (50 or 100 µM) or Alexa Fluor 594 hydrazide (20, 40 or 60 µM) as sodium salts (Invitrogen/Molecular Probes). The osmolality of this intracellular solution was ~290 mOsm/kg. Theoretical liquid junction potentials were calculated with JPCalcW (Axon Instruments, Union City, CA, USA) and we corrected all holding potentials for the liquid junction potential, either on-line via the data acquisition software (PatchMaster; HEKA Elektronik, Lambrecht/Pfalz, Germany) or off-line via direct subtraction.

### Electrophysiological recording and data acquisition

Patch pipettes were pulled from thick-walled borosilicate glass (outer diameter, 1.5 mm; inner diameter, 0.86 mm). Whole-cell voltage clamp recordings from AII amacrine cells were performed either with a conventional continuous single-electrode voltage-clamp (CSEVC; “patch clamp”) amplifier (EPC10-USB-dual or EPC10-triple; HEKA Elektronik) or with a discontinuous (switched) single-electrode voltage-clamp (DSEVC) amplifier (SEC-05LX-BF; npi Electronic, Tamm, Germany). All amplifiers were controlled by PatchMaster software. For recordings with a CSEVC amplifier, the open-tip resistance of the pipettes ranged from ~7 to ~12 MΩ when filled with intracellular solution. After establishing a GΩ-seal, currents caused by the recording electrode capacitance were automatically measured and neutralized by the amplifier. After breaking into the cell, currents caused by the cell membrane capacitance were partially neutralized by the amplifier. For recordings with a DSEVC amplifier, we used high-resistance pipettes with long, thin tips (open tip resistance ranged from ~25 to ~35 MΩ when filled with intracellular solution). For DSEVC amplifiers, the switching frequency (between current injection and potential measurement) was set to 35–40 kHz (duty cycle 1/4). The voltage-clamp gain and the proportional–integral controller were adjusted to give the fastest possible voltage response with minimal overshoot and ringing. The application of voltage commands and digital sampling of the analog signals were performed by an LIH8 + 8 laboratory interface (HEKA Elektronik; for a detailed description, see Veruki et al. 2008). During image acquisition, cells were voltage clamped at a holding potential of −60 mV. The sampling interval was set to 100 µs and before sampling, signals were low-pass filtered (CSEVC: analog three- and four-pole Bessel filters in series; DSEVC: analog four-pole Bessel filter) with a corner frequency (−3 dB) of 2–4 kHz. Two DSEVC amplifiers operated by two instances of PatchMaster running on the same computer were used for simultaneous recording and data acquisition from pairs of synaptically connected rod bipolar cells and AII amacrine cells.

### MPE microscopy and image acquisition

For MPE microscopy, fluorescence from neurons filled with Alexa 488 or 594 was imaged with the MOM equipped with a mode-locked Ti:sapphire laser (Mai Tai DeepSee; SpectraPhysics, Irvine, CA, USA) tuned to 775 nm for Alexa 488 and to 810 for Alexa 594. In a few experiments, we imaged simultaneously from pairs of cells filled with Alexa 488 and Alexa 594 and tuned the laser to either 775 or 810 nm. Scanning was performed by galvanometric scanners (XY, Cambridge Technology, Cambridge, MA, USA) with 3 mm mirrors. For increased spatial resolution, the laser beam was expanded to overfill the back aperture of the objective. Fluorescence and IR laser light were detected by separate multialkali photomultiplier tubes (R6357, Hamamatsu Corp.; Bridgewater, NJ, USA) and the analog signals were digitized by an acquisition board (NI-6110E, National Instruments, Austin, TX, USA). The intensity of the laser was attenuated and controlled by an electro-optic modulator (350-80LA with BK option; ConOptics, Danbury, CT, USA) driven by a 302RM amplifier (ConOptics). During image acquisition, exposure to laser light was controlled by an electronic shutter (LS6ZM2, Vincent Associates, Rochester, NY, USA), thereby minimizing the total exposure time. An image stack was acquired as a series of optical slices (each slice 1024 × 1024 pixels). To obtain well-sampled image stacks that could be processed with deconvolution (see “Image processing and deconvolution”), images were sampled at a rate close to the ideal Nyquist rate. The Nyquist sampling distance in the lateral direction was calculated as:

$$ \Delta_{x} = \Delta_{y} = \frac{{\lambda_{\text{ex}} }}{4kn \times \sin \alpha } $$

and for the axial direction, the Nyquist sampling distance was calculated as:

$$ \Delta_{z} = \frac{{\lambda_{\text{ex}} }}{{2kn \times \left( {1 - \cos \alpha } \right)}} $$

where *n* is the lens medium refractive index (1.338 for water), *k* is the number of excitation photons (photon count; set to 2 for MPE microscopy), *λ*_ex_ is the wavelength of the excitation light, and *α* is the half-aperture angle of the objective (reviewed by Heintzmann 2006; see also https://svi.nl/NyquistRate). For the majority of cells, the *XY* pixel size was ~72 or ~83 nm (depending on the digital zoom) and the focal plane interval (*Z*) was set to 0.4 µm, sufficient to satisfy Nyquist rate sampling according to the stated equations. At each focal plane, two or three images were averaged on-line to improve the signal-to-noise ratio (SNR). For each image stack, we acquired two or three channels. The first one or two channels sampled the fluorescence light as described above. The last channel was used for IR laser scanning gradient contrast (IR-LSGC) imaging (Yasuda et al. 2004) and sampled the forward scattered IR laser light after it passed the substage condensor and a Dodt gradient contrast tube (Luigs & Neumann). MPE microscopy and image acquisition were controlled by ScanImage software (version 3.7 or 3.8.1; Pologruto et al. 2003) running under Matlab (MathWorks, Natick, MA, USA).

### Image processing and deconvolution

The image stacks were de-interleaved based on acquisition channels (IGOR Pro, version 6 64-bit, WaveMetrics, Lake Oswego, OR, USA) and saved as individual files (one per channel). Huygens Essential (version 4 64-bit, Scientific Volume Imaging, Hilversum, The Netherlands) was used to remove noise and reassign out-of-focus light with a theoretically calculated point spread function, using the classic maximum likelihood estimation (CMLE) deconvolution algorithm. In addition, the object stabilizer module of Huygens Essential was used to align images along the *Z-*axis to compensate for drift and other mechanical instabilities. Processed image stacks were saved in 16-bit TIFF format, utilizing the whole dynamic range.

### 3D morphological reconstruction and measurements

Quantitative morphological reconstruction of the fluorescently labeled cells was done manually using computer-aided neuronal tracing software (Neurolucida; version 11 64-bit; MBF Bioscience, Williston, VT, USA; Glaser and Glaser 1990). 3D reconstruction of the soma was performed by tracing it with multiple contours at a series of different focal planes corresponding to different slices of the image stack. The surface area of the 3D reconstructed cells was calculated with the computer program Neurolucida Explorer (version 11 64-bit, MBF Bioscience). For general morphological analysis and quantification of dendritic branching metrics we used Neurolucida Explorer, L-measure (Scorcioni et al. 2008) and custom software written in IGOR Pro. We only imaged live cells, eliminating the need to correct for errors related to shrinkage. Cells displaying signs of mechanical injury or phototoxicity were not included in the material for reconstruction.

A dendritic varicosity was defined as a spatially discrete swelling where the maximum diameter increased ≥80 % relative to the diameter immediately before and after the swelling as visualized in the *XY* plane. Detection of varicosities was done manually in Neurolucida by visual inspection of the complete reconstruction by following it from soma to all endings. We used the “marker” functionality of Neurolucida to indicate the size and location (*XYZ*) of each varicosity, determined as the diameter and location (*XY*) of the largest circle that would fit inside the varicosity. The location in *Z* was determined by the reconstruction point corresponding to the largest diameter of the varicosity. After detection, the 3D viewer of Neurolucida was used to verify that no markers had been missed or misplaced along the *Z* axis. Subsequently, all varicosities were attached to the corresponding dendritic tree (using appropriate functions in Neurolucida) to enable analysis relative to branch order.

### Statistical analysis and data presentation

Data are presented as the mean ± SD (*n* = number of cells). Statistical analyses with comparisons between or within groups were performed using Student’s two-tailed *t* test (unpaired except where indicated). Differences were considered statistically significant at the *p* < 0.05 level. The number of individual traces included in the figures is stated for each case.

### Principal component and cluster analysis

To explore the homogeneity of the population of cells reconstructed, including the possible existence of subclasses or underlying variability, we performed principal component analysis (PCA) and cluster analysis (Matlab). For this analysis, all morphological metrics for each cell were included, as well as the total process length for each retinal layer and stratum (of the inner plexiform layer).

To avoid artificial weighting of properties, the raw data for each metric were centered on their means and normalized to their SD. Then, metrics that were largely redundant were excluded from the PCA and clustering. A metric was considered redundant when it displayed both a strong (positive or negative) correlation with and a clear geometrical relation to any other metrics. A strong correlation was defined as ∣correlation coefficient∣ >0.80 (Tsiola et al. 2003). For example, the number of branch segments was excluded for being redundant with the number of nodes (branch points). In addition, metrics that had practically no variation over the population were excluded to prevent the introduction of noise. An example is the average bifurcation angle, which was approximately 90° for all cells. For these excluded metrics, we calculated the regression coefficients with the PCs. In total, 27 metrics were included in the PCA.

Clustering was performed on the normalized data with Ward’s method (Ward 1963), as implemented in Matlab’s “linkage” and “clusterdata” functions. The “pca” function in Matlab was used to calculate the principal component decomposition and we obtained the principal components, their eigenvalues and the decomposition of each cell’s data into these components. The part of the data variance that a given principal component accounts for, similar to the coefficient of determination (*R* ^2^) of a simple linear regression, was calculated as the eigenvalue of the principal component divided by the number of included metrics. To assess significance of the principal components (Jackson 1993, and references therein), they were compared with those of a dataset containing randomly generated, normally distributed values (broken stick method). We then used bootstrap analysis to decide which individual metrics constituted statistically significant components of each principal component. For this, PCA was performed on 10,000 datasets of the same size, randomly sampled with repetitions from the original dataset. A *z* score was assigned, calculated as the absolute value of a metric’s coefficient in the principal component divided by its standard deviation obtained from the bootstrap analysis. In general, care should be taken that the (arbitrary) signs of the bootstrapped principal components are consistent with those from the original set and that the original order of the principal components is retained (Babamoradi et al. 2013). We corrected the signs by multiplying with the signs of the scalar products of the original and the bootstrapped principal components and checked that reordering was unnecessary.

## Results

### Visual targeting and identification of AII amacrine cells in retinal slices

**Fig. 1:**
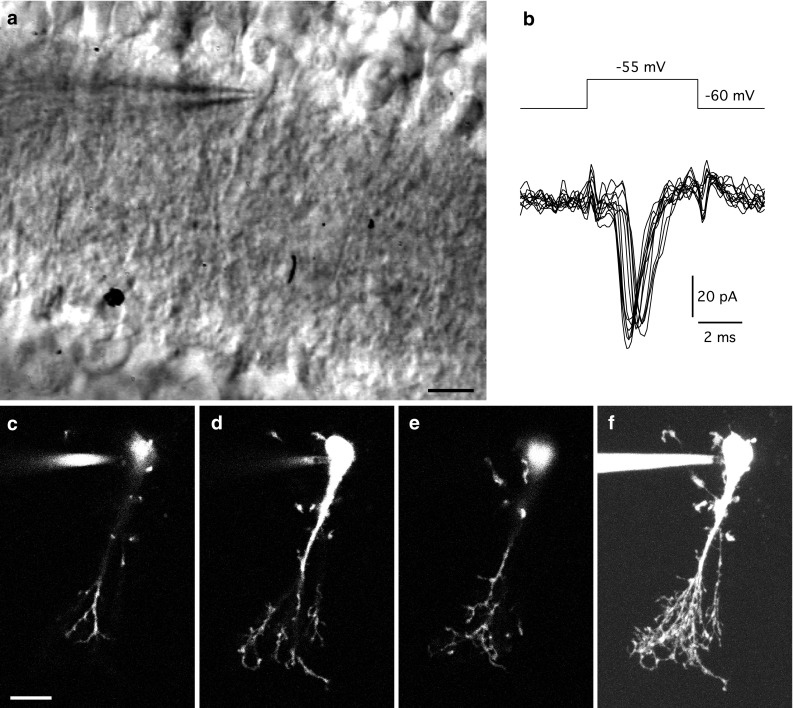
Visual targeting, electrophysiological recording and multi-photon excitation (MPE) microscopic live imaging of AII amacrine cells in retinal slices. **a** Infrared (IR) Dodt gradient contrast videomicrograph of an AII amacrine cell in a retinal slice. Cell body of AII amacrine visible at border between inner nuclear and inner plexiform layers. Tip of recording pipette located at cell body. Apical dendrite of AII amacrine visible as it descends into the inner plexiform layer. **b** Electrophysiological “signature” of AII amacrine cell (in **a**) during whole-cell voltage clamp recording (holding potential −60 mV). Transient inward currents (*bottom traces*) correspond to unclamped action currents (escape from voltage clamp) evoked by 5 mV depolarizing voltage pulses (*top trace*). **c**–**e** Individual image slices acquired with MPE microscopy after filling AII amacrine cell (in **a**) with the fluorescent dye Alexa Fluor 594. Separation between focal planes in **c** and **d** was 4.0 µm and between focal planes in **d** and **e** was 5.6 µm. Each image slice is the average of two individual frames. **f** Maximum intensity projection of complete image stack of the AII amacrine cell (total of 111 image slices separated by 0.4 µm). *Scale bars* 10 µm (**a**, **c**–**f**)

To ensure that only AII amacrine cells were included among the cells to be imaged and reconstructed, two main criteria had to be met during targeting in retinal slices. First, we took considerable care to only record and fill cells that displayed the morphological characteristics of AII amacrines, as judged by their appearance in retinal slices imaged with IR-DGC videomicroscopy (Fig. 1a). Specifically, the morphological criteria were the shape and location of the cell body at the border of the inner nuclear layer and the inner plexiform layer and the presence of a thick apical dendrite descending into the inner plexiform layer (Fig. 1a). Because we were interested in adapting and extending our workflow to include not only morphological reconstruction, but physiological recording and compartmental modeling as well, we filled the cells with fluorescent dyes via diffusion from patch pipettes instead of iontophoresis from sharp microelectrodes that are typically used for microinjection in live or fixed tissue. Second, by recording physiological responses immediately following the establishment of the whole-cell configuration, we verified that 5 mV depolarizing test pulses (5 ms duration, from a holding potential of −60 mV) evoked the characteristic inward action currents corresponding to unclamped action potentials (Fig. 1b) that depend on TTX-sensitive voltage-gated Na^+^ channels (Mørkve et al. 2002; Veruki et al. 2003). In previous studies, we have found that when cells are visually targeted in retinal slices according to this description, and subsequently display the characteristic action currents, they can always be positively identified as AII amacrine cells when examined with fluorescence microscopy (Mørkve et al. 2002; Veruki et al. 2003). Only cells that satisfied both these morphological and physiological selection criteria have been included in the material reported here.

After establishing the whole-cell recording configuration, we switched the optical pathway from IR-DGC videomicroscopy to MPE fluorescence microscopy. Focusing through the tissue allowed us to immediately verify the morphology of the cell as an AII amacrine cell (Fig. 1c–e). In addition, the forward scattered IR laser light enabled us to use IR-LSGC imaging to acquire contrast-enhanced images of the neuronal tissue in parallel with the MPE microscopic imaging. On-line overlay of the fluorescence and IR-LSGC images (in perfect register with each other) allowed us to verify the localization of the various subcellular structures of an AII amacrine cell at different levels of the inner plexiform layer (see Fig. 3a below). Acquisition of an image stack typically started approximately 10 min after establishing the whole-cell recording configuration and a complete stack required 25–35 min, depending on the number of slices and the number of frames averaged for each slice. During acquisition of a stack, a maximum intensity projection was calculated and continuously updated (Fig. 1f). In some cases, one or more additional stacks were sampled to take advantage of the enhanced fluorescence intensity obtained after a longer period of filling the cell with dye. The physiological condition of each cell was monitored by recording the holding current and input resistance throughout the acquisition period. In total, 43 AII amacrine cells (obtained from 26 different animals) were selected for digital reconstruction and quantitative morphometric analysis. At the holding potential of −60 mV, the average holding current was −12 ± 10 pA (range −40 to 7 pA, *n* = 43 cells) and the average input resistance was 721 ± 394 MΩ (range 260–2079 MΩ, *n* = 40 cells; the three additional cells were recorded in the presence of meclofenamic acid which blocks gap junctions and increases the input resistance; cf. Veruki and Hartveit 2009). The range of input resistances can most likely be explained by differences in the extent and conductance of gap junction coupling (cf. Veruki et al. 2010).

### Deconvolution of fluorescence image stacks

**Fig. 2:**
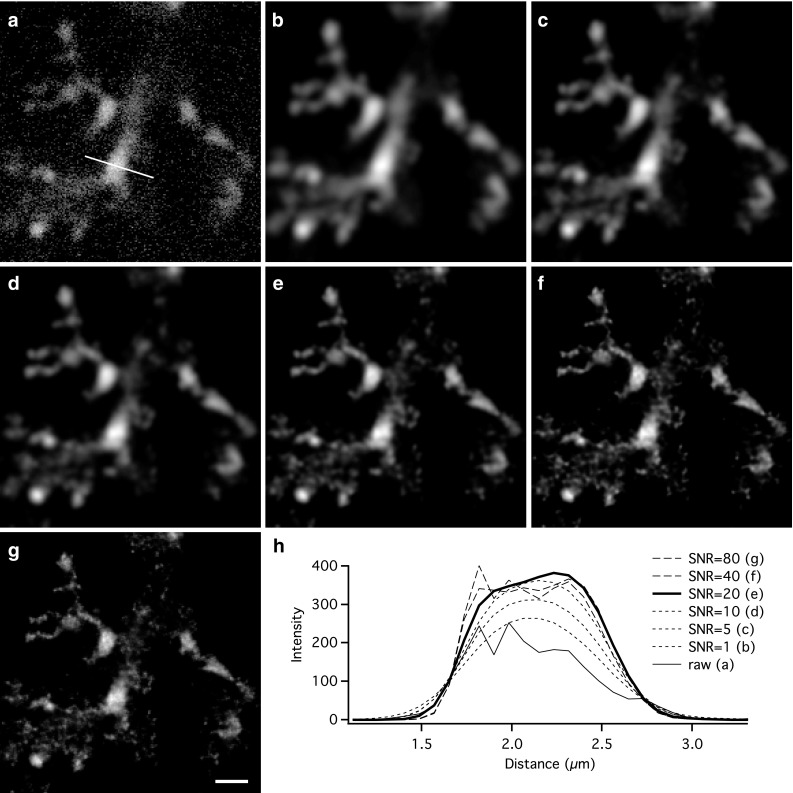
Procedure for digital deconvolution of MPE microscopic images of dye-filled AII amacrine cells. **a** Subregion of individual image slice (average of two individual frames) with details of arboreal dendrites of an AII amacrine cell. *Straight line* (length 4.4 µm) across process used to create intensity profiles displayed in **h**. Notice how image is affected by noise and blurring. **b**–**g** Same image as in **a** after deconvolution with different settings for the signal-to-noise ratio (SNR) in the deconvolution software, as indicated in **h**. Notice how deconvolution reduces noise and blurring and how increasing the SNR progressively improves the images, but eventually leads to spatial fragmentation (most pronounced in **f**, **g**). **h** Spatial intensity profiles of raw image (**a**) and deconvolved images (**b**–**g**) for different values of SNR during deconvolution. Notice noisy profile from raw image, reaching a peak intensity of approximately 200 (*thin continuous line*) and how increasing the SNR (**b**–**d**; *broken smooth lines*) increases the peak intensity from approximately 250 to approximately 350. For SNR of 20 (**e**), the intensity profile reaches an overall maximum while still remaining relatively smooth (*thick continuous line*). For SNRs of 40 (**f**) and 80 (**g**), the profiles become noisy, corresponding to spatial fragmentation seen in the images (**f**, **g**). Brightness, contrast and gamma settings were identical for **a**–**g**. *Scale bar* 2 µm (**a**–**g**)

Digital deconvolution is a powerful post-acquisition computer image processing technique for enhancing image quality (Cannell et al. 2006; Murphy and Davidson 2013). Before digital morphological reconstruction, each fluorescence image stack was deconvolved to increase the SNR and decrease the axial and lateral blurring (van der Voort and Strasters 1995). The deconvolution software (Huygens Essential) requires user input of several microscopic and imaging parameters whereas the default values of other parameters are calculated from the data in the image stack. One user-specified parameter, the SNR, controls the sharpness of the restoration result, but can lead to enhanced noise when it is set higher than an optimal value. For each image stack, we estimated an optimal SNR by repeating the deconvolution for several values of SNR while keeping all other parameters and settings constant. Figure 2 illustrates an example of the results obtained by this procedure for dendrites in an arbitrary region within a single focal plane of an AII amacrine image stack. Figure 2a shows raw image data and Fig. 2b–g show results after deconvolution with different SNRs (set to 1, 5, 10, 20, 40, and 80, respectively). Deconvolution with increasing SNR increases the sharpness of the images and removes noise corresponding to out-of-focus light, but when the SNR of the deconvolution procedure is increased to 40 and 80 (Fig. 2f, g), the resulting images display clear structural fragmentation of the dendrites, indicating that the SNR values were too high. We analyzed this in more detail by plotting the intensity profiles for fluorescence across different dendritic processes. For the example in Fig. 2, the intensity profiles were calculated along the line displayed in Fig. 2a. Figure 2h shows the results for the original image and the images generated by deconvolution for a range of SNRs (Fig. 2b–g). Deconvolution with increasing values for the SNR progressively increased the peak value of the intensity profile, but when the SNR was increased above the optimal value (approximately 20 in the example of Fig. 2), the intensity profile and the corresponding image displayed increasing noise and morphological fragmentation, respectively. To ensure optimal processing, we applied this procedure and analyzed several regions of the stack for all cells reconstructed.

### Quantitative morphological reconstruction

**Fig. 3:**
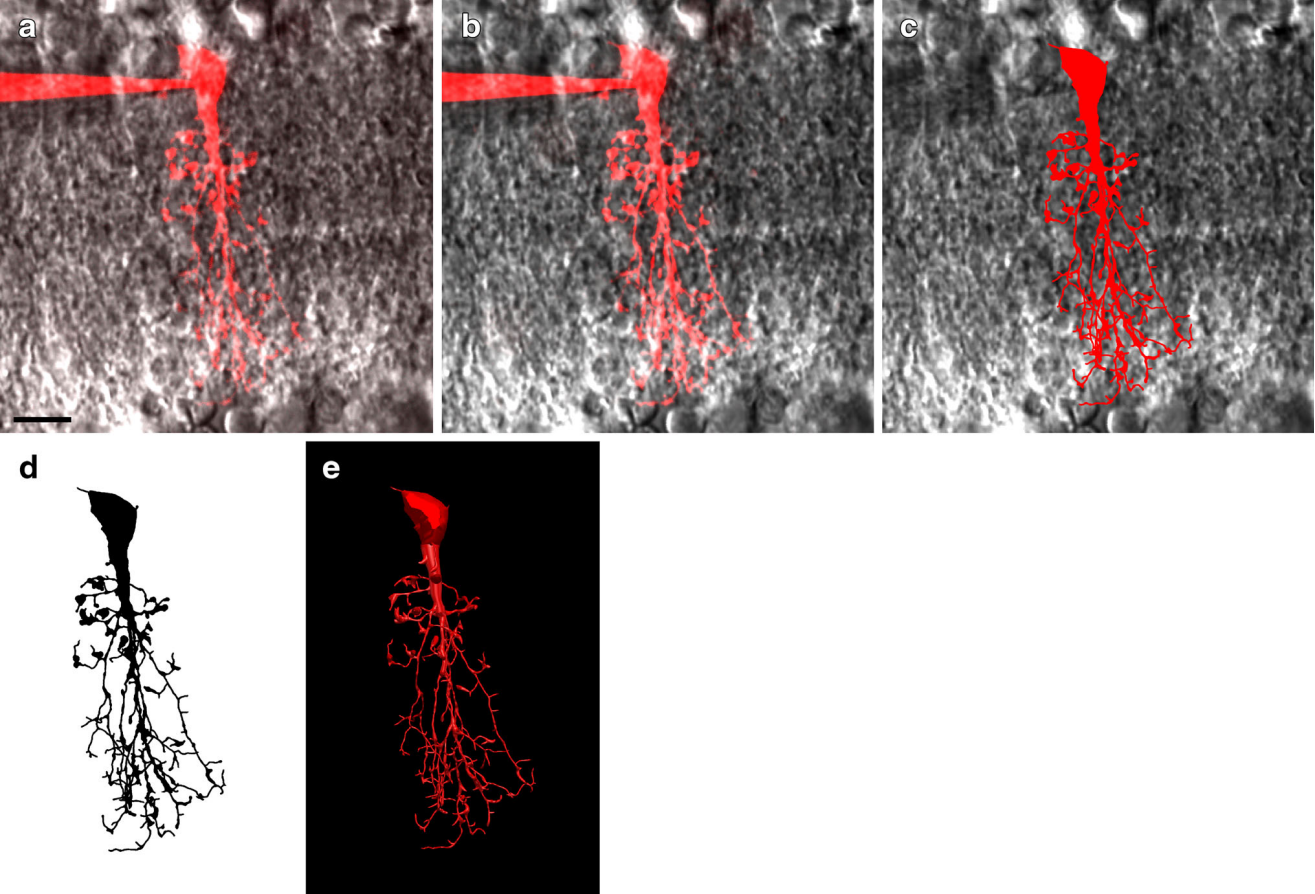
Workflow for MPE microscopic imaging and quantitative morphological reconstruction of dye-filled AII amacrine cells. **a** Maximum intensity projection of raw image stack of AII amacrine cell filled with Alexa Fluor 594 during whole-cell recording (dye-filled pipette attached to the cell body) overlaid on image of retinal slice acquired with IR-laser scanning gradient contrast microscopy. **b** Same as in **a**, but after deconvolution. **c** Shape plot generated by computerized morphological reconstruction of cell in **a** and **b**. Brightness and contrast of background image of retina had to be re-adjusted for composite images in **a**–**c**. **d** Shape plot of reconstructed cell showing details of dendritic arborization. **e** Three-dimensional (3D) view of morphological reconstruction. *Scale bar* 10 µm (**a**–**d**)

Prior to quantitative morphological analysis we performed accurate digital reconstruction, a prerequisite for quantitative morphometry and the extraction of a series of morphological measures. Each cell was reconstructed by manually tracing the fluorescent processes through the image stack, using Neurolucida. Figure 3a–c illustrates three different stages of the reconstruction workflow, with maximum intensity projections of the fluorescence image stack before (Fig. 3a) and after (Fig. 3b) deconvolution, and a projection of the final digital reconstruction (Fig. 3c). All projections have been overlaid on a single, representative image slice from the IR-LSGC channel (identical for panels a–c). The details of the dendritic arborization of the reconstructed neuron are more clearly displayed by the two-dimensional (2D) projection (shape plot) in Fig. 3d and the 3D visualization in Fig. 3e.

For quantitative morphological reconstruction and analysis based on light microscopic imaging, it is a problem when the diameters of the thinnest neuronal processes are below the resolution limit of light microscopy (Jaeger 2001; Jacobs et al. 2010). For self-luminous point objects, as in fluorescence light microscopy, the lateral (*XY*) Rayleigh two-point resolution (minimum resolved distance) is given by 0.61*λ*/*N*_A_ (e.g. Murphy and Davidson 2013; Wouterlood and Beliën 2014), where *λ* is the wavelength of the light and *N*_A_ is the numerical aperture of the microscope objective. With MPE microscopy, where only the excitation wavelength is important, the resolution is improved by $\sqrt 2$ (in the ideal, diffraction-limited case, assuming that the laser beam completely fills the back-focal plane of the objective) and the equation becomes $0.61\lambda /(N_{\text{A}} \sqrt 2 )$ (Cox and Sheppard 2004). With an excitation wavelength of 810 nm and *N*_A_ = 0.95 for the objective used, the resolution limit becomes approximately 0.37 µm in the ideal (diffraction-limited) case. This means that when processes are thinner than this, they can be detected if the intensity is sufficiently high, but the diameter cannot be adequately resolved. Currently, electron microscopy is the only reliable source of information when the diameters of the thinnest processes of a specific type of neuron are below the light microscopic resolution limit. Unfortunately, even though AII amacrine cells from several species, including rat, have been investigated at the ultrastructural level, there is a lack of detailed information in the scientific literature. A notable exception with direct relevance for our study is a recent report of AII amacrine cells in mouse retina that illustrated 2D projections of complete electron microscopic reconstructions of three AII amacrine cells (Tsukamoto and Omi 2013). By making measurements from the thinnest processes illustrated (their Fig. 1), we estimated the diameter of these processes to be 0.23 ± 0.05 µm (range 0.14–0.31 µm; *n* = 31 diameters; 10–11 measurements for each of three cells). This is clearly below the expected resolution limit of our MPE imaging system. Accordingly, all morphological reconstructions were digitally corrected with the following procedure. First, for each reconstruction we averaged the diameters of the 10 thinnest reconstruction points (on 10 unique branch segments). The difference between 0.23 µm and this average was added to all process diameters such that the average of the ten thinnest reconstruction points became 0.23 µm. For most cells, the diameters of a reconstruction were increased, typically by approximately 0.1 µm. For one cell, the diameters were corrected by subtraction of 0.04 µm. Apart from short terminal branches, we consider it unlikely that this problem of resolution contributed to underestimating the number and total length of dendritic branches of the AII amacrine cells. Because the large majority of AII processes contain one or more thicker varicosities along or at the termination, a human operator usually has no problem correctly identifying and connecting daughter branches to parent branches during reconstruction.

### Qualitative morphological characteristics of AII amacrine cells

**Fig. 4:**
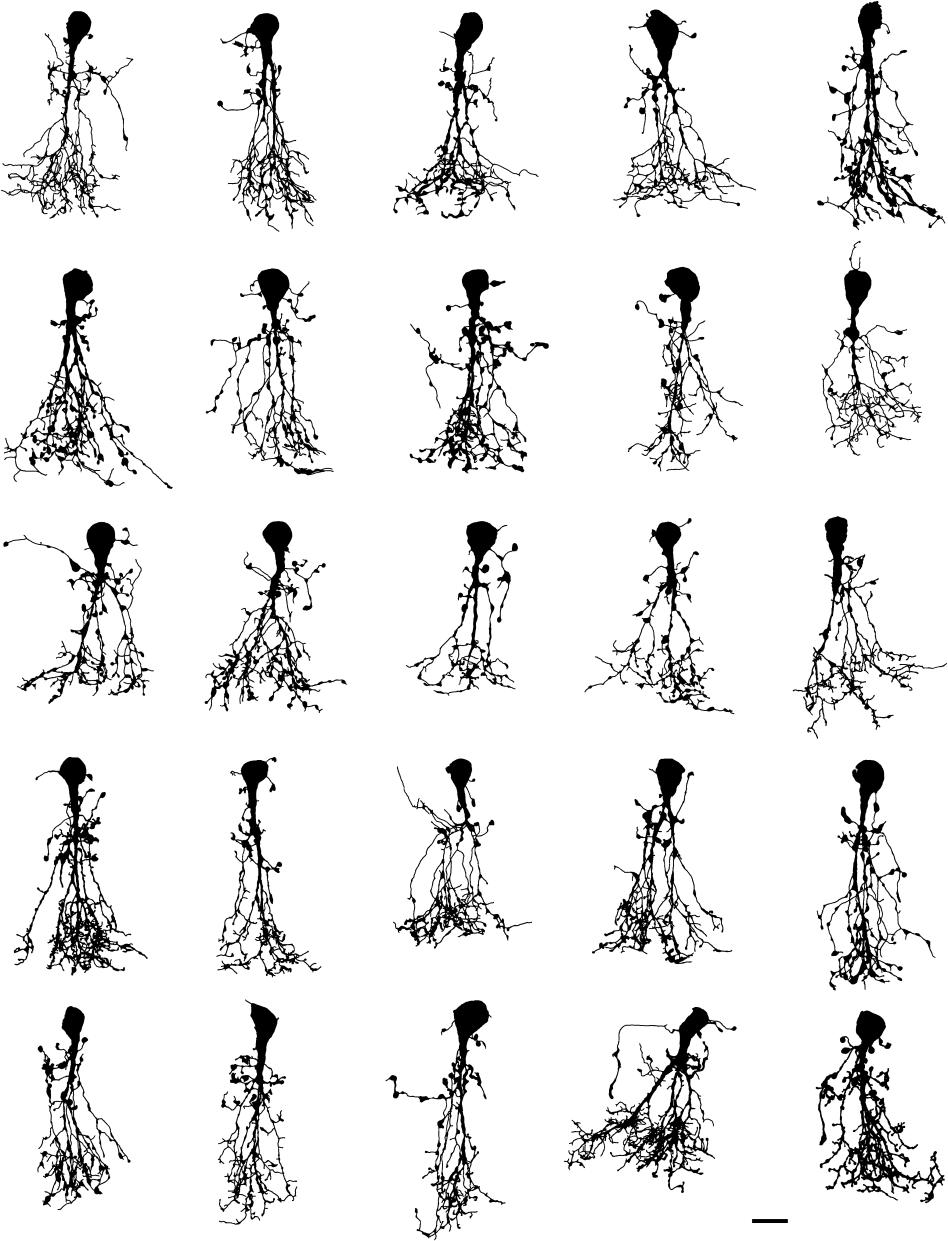

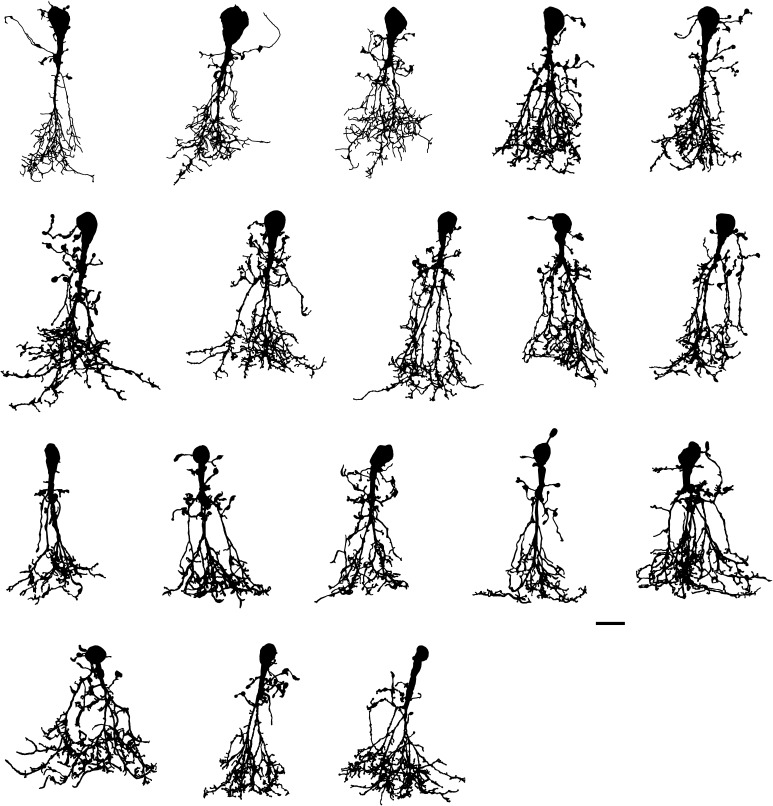
Shape plots of all morphologically reconstructed AII amacrine cells (*n* = 43). All cells were filled with fluorescent dye by whole-cell recording in retinal slices, imaged with MPE microscopy and morphologically reconstructed. Notice common morphological properties as well as considerable heterogeneity. Cells have been rotated in the *XY* plane as required to orient the long axis vertically. *Scale bars* 10 µm

The total population of AII amacrine cells morphologically reconstructed from fluorescent image stacks acquired with MPE microscopy is illustrated by shape plots, corresponding to projections in the *XY* plane, in Fig. 4. Despite morphological variability, these cells display a set of common characteristics that together contribute to defining them as a cell type. AII amacrine cells have been characterized as axon-less, narrow-field, bistratified retinal interneurons and their general morphological characteristics have been identified in a variety of different mammalian species at the light microscopic level, including cat (Kolb and Famiglietti 1974; Famiglietti and Kolb 1975; Kolb et al. 1981; Vaney 1985), dog (Cajal 1892; Famiglietti and Kolb 1975), mouse (Wu et al. 2011; Cembrowski et al. 2012), primate (Polyak 1941; Boycott and Dowling 1969; Famiglietti and Kolb 1975; Kolb et al. 1992; Wässle et al. 1995), rabbit (Dacheux and Raviola 1986; Mills and Massey 1991; Vaney et al. 1991), and rat (Perry and Walker 1980; Boos et al. 1993; Wässle et al. 1993; Mørkve et al. 2002). The number of studies of AII amacrine cells at the electron microscopic level is smaller, but includes cat (Kolb and Famiglietti 1974; Famiglietti and Kolb 1975; Kolb 1979), mouse (Tsukamoto and Omi 2013), primate (Wässle et al. 1995), rabbit (Dacheux and Raviola 1986; Strettoi et al. 1992; Marc et al. 2014), and rat (Chun et al. 1993).

**Fig. 5:**
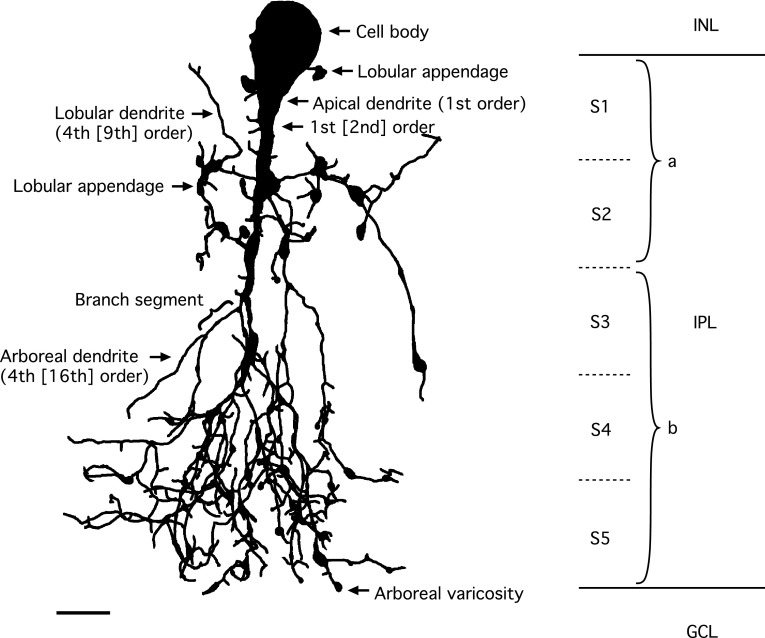
Shape plot of morphologically reconstructed AII amacrine cell and nomenclature used to describe branching and branch ordering. Shape plot illustrates characteristic features of AII cellular morphology with shape of and relationships between cell body, apical dendrite and lobular and arboreal dendrites. “Branch segment” illustrates definition of segment between two points of arborization. Notice varicosities in the form of lobular appendages and arboreal varicosities. Shape plot also indicates a few examples of segment branch orders (first- and fourth-order dendritic branches) resulting from a central shaft branch ordering scheme, with corresponding branch orders for the same segments resulting from a centrifugal branch ordering scheme in *square brackets*. The borders between retinal layers and strata are marked at *right*. The retinal layers are indicated by abbreviations (*INL* inner nuclear layer, *IPL* inner plexiform layer, *GCL* ganglion cell layer) and the IPL has been divided into five equally thick strata (stratum 1 (S1)–S5), with S1–S2 corresponding to sublamina *a* and S3–S5 corresponding to sublamina *b*. *Scale bar* 5 µm

The bistratified dendritic morphology of AII amacrine cells corresponds to distinct arborizations in sublamina *a* (corresponding to S1 and S2 when the inner plexiform layer is divided into five equally thick strata; S1–S5) and sublamina *b* (corresponding to S3, S4 and S5) of the inner plexiform layer (Fig. 5). The cells typically have a single, thick apical dendrite that descends from the cell body and tapers as it runs vertically into sublamina *a* and branches into a number of arboreal dendrites in sublamina *b*, collectively referred to as a conical arborization (Figs. 4, 5). In addition, AII amacrines have a number of thinner processes in sublamina *a* termed lobular dendrites (Figs. 4, 5). These spread laterally and can arise in one of three different ways; directly from the soma, directly from the apical dendrite or (indirectly) from a proximal location (relative to its origin from the soma) of an arboreal dendrite (Fig. 4). The latter type of lobular dendrites were termed isolated lobular dendrites for AII amacrines in cat retina (Vaney 1985). Along their course, the lobular dendrites can carry large varicosities and upon termination they often swell into a large, irregular varicosity, all of which are referred to as lobular appendages (Figs. 4, 5). Most of the lobular appendages seem to be clustered in a relatively tight field or volume close to the cell body and apical dendrite. However, it is also possible to observe that AII amacrines can give rise to a lobular dendrite extending considerably beyond the main area occupied by lobular dendrites and appendages (Fig. 4). This lobular dendrite is likely to correspond to the process identified as displaying a cluster of voltage-gated Na^+^ channels (Wu et al. 2011; Cembrowski et al. 2012) and having a characteristic ultrastructure in electron microscopic investigations (Tsukamoto and Omi 2013). It is also possible to observe that lobular dendrites can extend into the inner nuclear layer (Fig. 4), similar to what has been reported for AII amacrine cells in rabbit retina (Casini et al. 1995).

The arboreal dendrites in sublamina *b* typically arise from the branching apical dendrite, but they can also descend from processes that arise independently as lobular dendrites from the apical dendrite or directly from the cell body (Fig. 4). The arboreal dendrites arising as secondary branches from the thick apical dendrite further subdivide as they traverse the inner plexiform layer and either terminate or spread tangentially at the border between the inner plexiform layer and ganglion cell layer before terminating. Along their course, they can give rise to spiny projections or irregular, varicose swellings and toward their end, the branches of the arboreal dendrites can terminate abruptly or with a varicose swelling (Figs. 4, 5).

### Morphological relationship between AII amacrine cells and presynaptic rod bipolar cells

**Fig. 6:**
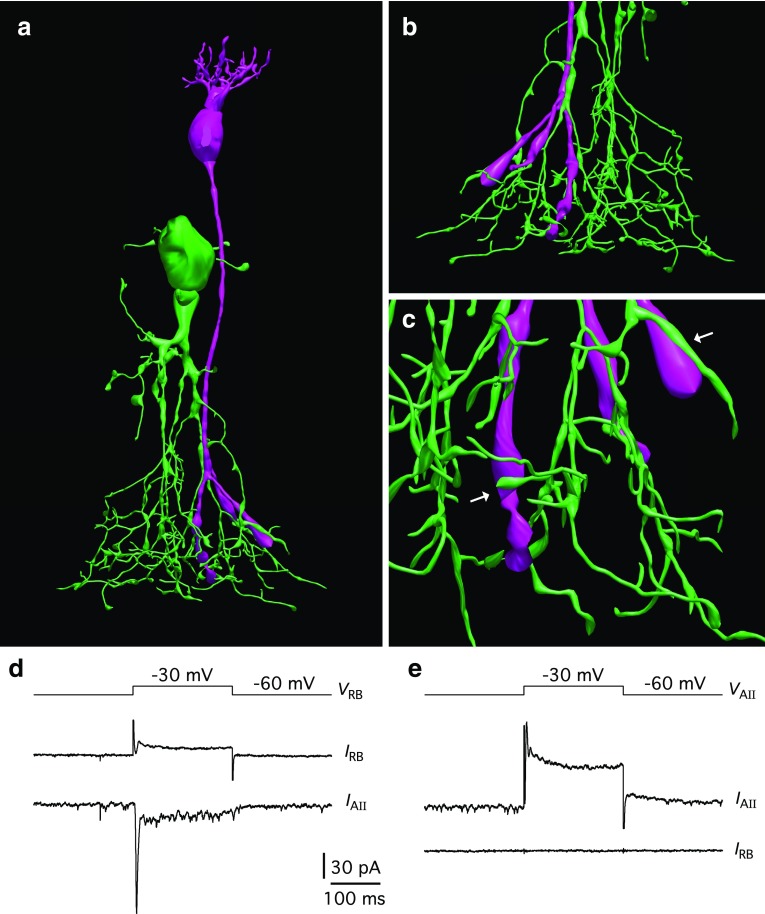
Morphological reconstruction of synaptically connected cell pair with presynaptic rod bipolar cell and postsynaptic AII amacrine cell. **a** 3D view of reconstructed rod bipolar cell (*magenta*) and AII amacrine cell (*green*). During whole-cell recording, the rod bipolar was filled with Alexa Fluor 594 and the AII amacrine was filled with Alexa Fluor 488 for MPE microscopic imaging. **b** Branching pattern of AII arboreal dendrites and rod bipolar axon terminals seen from the rear (opposite view from **a**). **c** Detailed view of close appositions between large axon terminals of rod bipolar and thinner arboreal dendrites of AII amacrine, potentially corresponding to synaptic contacts (*arrows*). **d**, **e** With simultaneous paired voltage-clamp recording, synaptic connectivity was verified by depolarizing the rod bipolar cell from the holding potential (−60 mV) to −30 mV (**d**, *top trace*; *V*_RB_), evoking an excitatory postsynaptic current in the AII amacrine cell (**d**, *bottom trace*; *I*_AII_). The current response of the rod bipolar (*I*_RB_) reflected a combination of depolarization-evoked voltage-gated Ca^2+^ and K^+^ currents (**d**, *middle trace*). When the AII amacrine cell was depolarized in the same way (**e**, *top trace*; *V*_AII_), this only evoked a response in the AII itself (**e**, *middle trace*; *I*_AII_), with no response in the rod bipolar (**e**, *bottom trace*; *I*_RB_). Capacitative current transients caused by the voltage steps have been truncated

For two AII amacrine cells, we recorded from and successfully reconstructed a presynaptic rod bipolar cell. Rod bipolar cells receive synaptic input from rod photoreceptors in the outer plexiform layer and provide chemical (glutamatergic) synaptic input to AII amacrine cells at their axon terminals in the proximal part (S5) of the inner plexiform layer (Kolb and Famiglietti 1974; Famiglietti and Kolb 1975; Strettoi et al. 1992; Singer and Diamond 2003; Tsukamoto and Omi 2013). For the reconstructed cell pair illustrated in Fig. 6a, the cell body of the rod bipolar cell was located in the distal part of the outer nuclear layer, with clearly visible dendrites in the outer plexiform layer, and only minimal lateral separation relative to the cell body of the AII amacrine cell. The long axon from the rod bipolar cell descended into the inner plexiform layer and divided into shorter terminal branches with several large swellings corresponding to axon terminals (Fig. 6a, b). Whereas light microscopic imaging cannot identify synaptic contacts as such, we observed several appositions where the relatively thin processes of the AII arboreal dendrites came in close contact with axon terminal swellings of the rod bipolar cell. The reconstruction of two such potential contacts is illustrated at higher magnification in Fig. 6c. During the recording, we verified the presence of synaptic connectivity between the two cells by alternately depolarizing each cell. Depolarizing the rod bipolar cell from a holding potential of −60 to −40 mV (or more positive), evoked a transient inward current in the AII amacrine cell, followed by a smaller sustained response component for the duration of the depolarization (Fig. 6d; cf. Singer and Diamond 2003). In contrast, when we depolarized the AII amacrine cell in the same way, only a response in the AII could be observed, with no response in the rod bipolar cell. This contrasts with the responses expected for pairs of AII amacrine and ON-cone bipolar cells which are connected by electrical synapses (Veruki and Hartveit 2002b). Similar morphological and physiological properties were observed for the other cell pair recorded. This result supports the identity of the recorded cells and their intact synaptic connectivity in our preparation.

### Quantitative morphological characteristics and branch ordering of AII amacrine cells

The standard description of single cell neuronal morphology of AII amacrine cells, as summarized above, typically provides only minimal and qualitative information concerning issues of variability and ignores quantitative aspects of several morphological parameters. For example, there is a paucity of quantitative data concerning both global and local properties of neuronal arborization, including branching pattern, dendritic lengths and diameters, surface area, and number and distribution of dendritic varicosities. Such data are important for studies that address questions of development, plasticity, and degeneration (Bernard et al. 2008; Bestman et al. 2008; Dunaevsky and Woolley 2008). The extensive branching of AII arboreal dendrites over a small volume can be described qualitatively, but must be supported by quantitative measurements.

After morphological reconstruction, quantitative morphometric analysis was performed using the computer programs Neurolucida Explorer and L-measure (Scorcioni et al. 2008), as well as custom software programmed in the IGOR Pro environment. For all 43 cells, we analyzed a series of geometric and topological parameters of neuronal morphology, summarized in Table 1 that shows the average values (±SD) and the ranges for all cell body and dendritic branching parameters. Soma volume and surface area were calculated from the multiple contours used to trace the cell body at a consecutive series of focal planes corresponding to individual slices. The projection of the cell body in the *XY* plane was used to calculate perimeter and Feret maximum and minimum. Dendritic length was calculated as the total length of all processes irrespective of the identity of the individual dendritic trees. A branch segment was defined as the part of a branch between two nodes (Fig. 5) or between a node and a termination point (ending; Capowski 1989). The number of segments equals the sum of the number of nodes and the number of termination points. The 2D convex hull (area) was measured separately for the part of the dendritic tree located in the proximal region of the inner plexiform layer (corresponding to the arboreal dendrites) and for the part of the dendritic tree located in the distal region of the inner plexiform layer (corresponding to the lobular dendrites). In both cases, the 2D convex hull was measured for the projection onto the *XZ* plane, i.e. the surface of the retina. The volume and surface area of the 3D convex hull were measured for the combination of all dendritic trees, excluding the cell body.

**Table 1:** Morphological properties of reconstructed AII amacrine cells

Parameter	Mean ± SD (*n* = 43)	Range
Soma volume (µm^3^)	280 ± 110	60 to 560
Soma surface area (µm^2^)	197 ± 67	68 to 338
Soma projection area (µm^2^)	44 ± 13	18 to 84
Soma projection perimeter (µm)	25.8 ± 4.3	16.3 to 37.1
Soma projection Feret maximum (µm)	9.1 ± 1.4	5.8 to 12.8
Soma projection Feret minimum (µm)	6.7 ± 1.1	4.3 to 9.4
Number of primary dendrites	3.4 ± 1.9	1 to 9
Length of main primary dendrite (µm)	12.9 ± 6.0	5.7 to 36.2
Maximum diameter of main primary dendrite (µm)^a^	3.06 ± 0.47	1.87 to 4.33
Dendritic length (µm)	1080 ± 270	500 to 1630
Dendritic surface area (µm^2^)	1770 ± 570	850 to 3160
Dendritic volume (µm^3^)	310 ± 110	140 to 620
Average dendritic diameter (µm)^a^	0.450 ± 0.070	0.307 to 0.600
Average branch segment path length (µm)^a^	3.19 ± 0.56	2.34 to 4.99
Maximum branch order (central shaft ordering)	22.8 ± 6.2	10 to 39
Maximum branch order (centrifugal ordering)	26.6 ± 6.8	12 to 40
Average partition asymmetry^a^	0.619 ± 0.038	0.502 to 0.687
Number of nodes	165 ± 63	46 to 311
Number of endings	178 ± 66	64 to 324
Number of varicosities	125 ± 40	59 to 268
2D convex hull area, arboreal dendrites (µm^2^)^b^	810 ± 270	400 to 1560
2D convex hull perimeter, arboreal dendrites (µm)^b^	108 ± 18	79 to 154
2D convex hull Feret max., arboreal dendrites (µm)^b^	40.4 ± 6.6	30.3 to 55.3
2D convex hull Feret min., arboreal dendrites (µm)^b^	27.9 ± 5.4	19.3 to 42.9
2D convex hull area, lobular dendrites (µm^2^)^b^	380 ± 140	140 to 690
2D convex hull perimeter, lobular dendrites (µm)^b^	78 ± 16	45 to 110
2D convex hull Feret max., lobular dendrites (µm)^b^	30.1 ± 6.9	15.0 to 42.9
2D convex hull Feret min., lobular dendrites (µm)^b^	18.0 ± 3.6	11.5 to 27.6
3D convex hull volume, dendritic tree (µm^3^)	27.0 ± 8.3 × 10^3^	11.2 to 62.9 × 10^3^
3D convex hull surface area, dendritic tree (µm^2^)	5160 ± 940	3080 to 8970
Euclidean distance from soma (mean) (µm)^a^	37.7 ± 2.9	31.0 to 43.1
Euclidean distance from soma (maximum) (µm)^a^	57.9 ± 4.4	49.5 to 69.0
Bifurcation angle (mean) (deg; Bif_ampl_remote)^a^	86.0 ± 3.0	79.9 to 94.0
Bifurcation angle (standard deviation)^a^	31.7 ± 2.4	26.7 to 36.4
Bifurcation tilt (mean) (deg; Bif_tilt_remote)^a^	104.8 ± 3.6	97.2 to 115.7
Bifurcation tilt (standard deviation)^a^	28.2 ± 2.2	22.2 to 33.7
Contraction^a^	0.887 ± 0.016	0.859 to 0.924
Fractal dimension^a^	1.0405 ± 0.0080	1.0203 to 1.0580
Helicity (mean)^a^	−0.1 ± 1.4 × 10^−3^	−2.8 to 3.9 × 10^−3^

Metrics were obtained from Neurolucida Explorer, except those marked with ^a^ from L-measure and ^b^ from custom IGOR Pro code. For some metrics where it would otherwise not be obvious, the L-measure function names are stated in parenthesis. For all metrics except Euclidean distance from soma, bifurcation angle and bifurcation tilt, each cell contributed one data point and the averages and SDs were calculated for the 43 data points

Average dendritic diameter: obtained by averaging the diameters of all reconstructed points. In general, the reconstruction points were fairly evenly spaced over a reconstructed dendritic tree, but the thickest, lower-order branches could be slightly under-represented

Average partition asymmetry: a measure for how much a neuronal tree deviates from a symmetrically partitioned tree where each node gives rise to two subtrees that contain an equal number of nodes, with 0 corresponding to a perfectly symmetric tree and 1 corresponding to a maximally uneven distribution of nodes, i.e. a tree containing a single long process with only single branches sprouting off

Euclidean distance from the soma: calculated for all reconstruction points as the Euclidean distance from the centroid of the soma. For each cell, we report the average (without weighting for the compartment length or diameter) and the maximum (showing how far from the soma the branches can extend)

Bifurcation angle: measures the angle between the two daughter branch segments of a bifurcation (angle measured between lines connecting the start and end points of the daughter segments)

Bifurcation tilt: measures the angle between the parent compartment and the daughter branch segment that sprouts the most backward (angle measured between a line connecting the last two reconstruction points of the parent branch segment and a line connecting the start and end points of the relevant daughter segment), with 180° corresponding to the forward direction and 0° corresponding to the backward direction

Contraction: measures the ratio between the Euclidean distance and the path distance of the end points of a branch segment

Fractal dimension: a measure of how much a branch resembles a fractal object (or random walk), with a value of 1 corresponding to a branch steadily growing in one direction and a value of 2 corresponding to a random walk

**Fig. 7:**
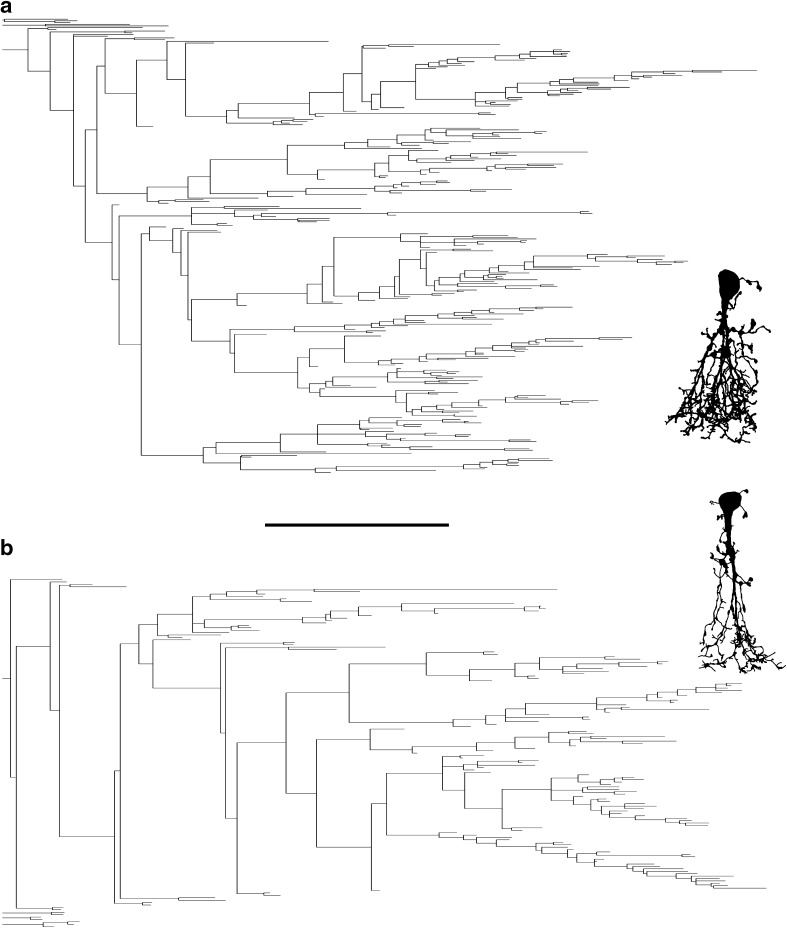
Dendritic tree diagrams (dendrograms) for AII amacrine cells. **a**, **b** Dendrograms for AII amacrines with relatively profuse (**a**) and sparser (**b**) branching (shape plot of reconstructed morphology in *insets* to the *right*). The length of each *horizontal line* in the dendrogram corresponds to the path length of each segment. The primary dendrites originating from the cell body are displayed to the *left*. Notice that for each cell, most of the branches arise from a single primary dendrite. *Scale bar* 20 µm (for the dendrograms)

From Table 1 it can be seen that although AII amacrines are typically dominated by a thick, vertically oriented apical dendrite, the average number of primary dendrites (1st order dendrites or “stems”) was 3.4, with a range from 1 to 9. The total number of nodes ranged from 46 to 311 and the maximum branch order ranged from 10 to 39 (with a central shaft branch ordering scheme; see below). These metrics are considerably higher than expected from previous descriptions and illustrations in the scientific literature, e.g. compared to published images based on Golgi impregnations (Perry and Walker 1980). The extensive branching of AII amacrine cells can be observed qualitatively by shape plots (Fig. 4) and is illustrated for two cells by dendrograms where the branching is easier to inspect (Fig. 7).

For AII amacrine cells, we found an average partition asymmetry close to 0.6 (Table 1). For our cells, this metric is primarily determined by the ratio (typically 2:3) between “tip bifurcations”, from which two single branches sprout that do not bifurcate any further (corresponding to a partition asymmetry of 0) and “stub bifurcations”, at which only one of the two daughter trees is a single branch (corresponding to a partition asymmetry of 1).

The average bifurcation angle was 86° and the average of the standard deviation was 32° (Table 1). These values correspond approximately to an even distribution of branch angles over a sphere, which can be calculated to have an average of 90° and a standard deviation of 39° (not shown; for the distribution density of angles on a sphere, see: Weisstein EW. Sphere point picking, at MathWorld, a Wolfram Web Resource. http://mathworld.wolfram.com/SpherePointPicking.html 2015 and references therein).

The average bifurcation tilt was 105° (Table 1), indicating that the branches of a typical AII amacrine have a tendency to sprout in the forward direction. For comparison, we calculated an average bifurcation tilt of 68° for evenly distributed, random branching (not shown). The average of the standard deviation for the bifurcation tilt of AII amacrines was 28°, similar to the expected value for random branching which we calculated to be 32°. This suggests that despite their preference for the forward direction, the branches do sprout in a wide range of angles.

The average values for contraction (0.89) and fractal dimension (1.04; Table 1) indicate that the dendrites of AII amacrine cells are not straight lines, but are slightly meandering, similar to what has been observed for dendrites of other neurons (e.g. Marks and Burke 2007). The branches of AII amacrine cells show negligible helicity, i.e. they do not grow in corkscrew trajectories (Table 1).

### Dendritic parameters as a function of segment branch order

For analysis of dendritic parameters as a function of segment branch order, we considered two different branch ordering schemes. With the centrifugal branch ordering scheme, each branch point leads to an increment of the branch order of both daughter segments, irrespective of their relative thickness. Although this scheme is unambiguous and in principle easy to interpret, its application to the AII amacrine can appear somewhat counterintuitive because this cell type is typically dominated by a thick apical dendrite (Fig. 5). A more natural alternative is to apply the central shaft branch ordering scheme. For an AII amacrine cell, this means that the branch order of the apical dendrite remains constant at 1 along its length and segments that branch off the apical dendrite are all assigned branch order 2. Figure 5 illustrates a few examples of the different branch orders assigned to segments when the two different schemes of branch ordering are applied to the same AII amacrine. With the central shaft branch ordering scheme, the maximum branch order ranged from 10 to 39, with a mean of 23 and with the centrifugal branch ordering scheme, the maximum branch order ranged from 12 to 40, with a mean of 27 (Table 1). Irrespective of the scheme, these values are considerably higher than the only previously published value for this parameter, reported as “higher than 8” by Sterling (1983) for cat AII amacrines reconstructed by electron microscopy.

**Fig. 8:**
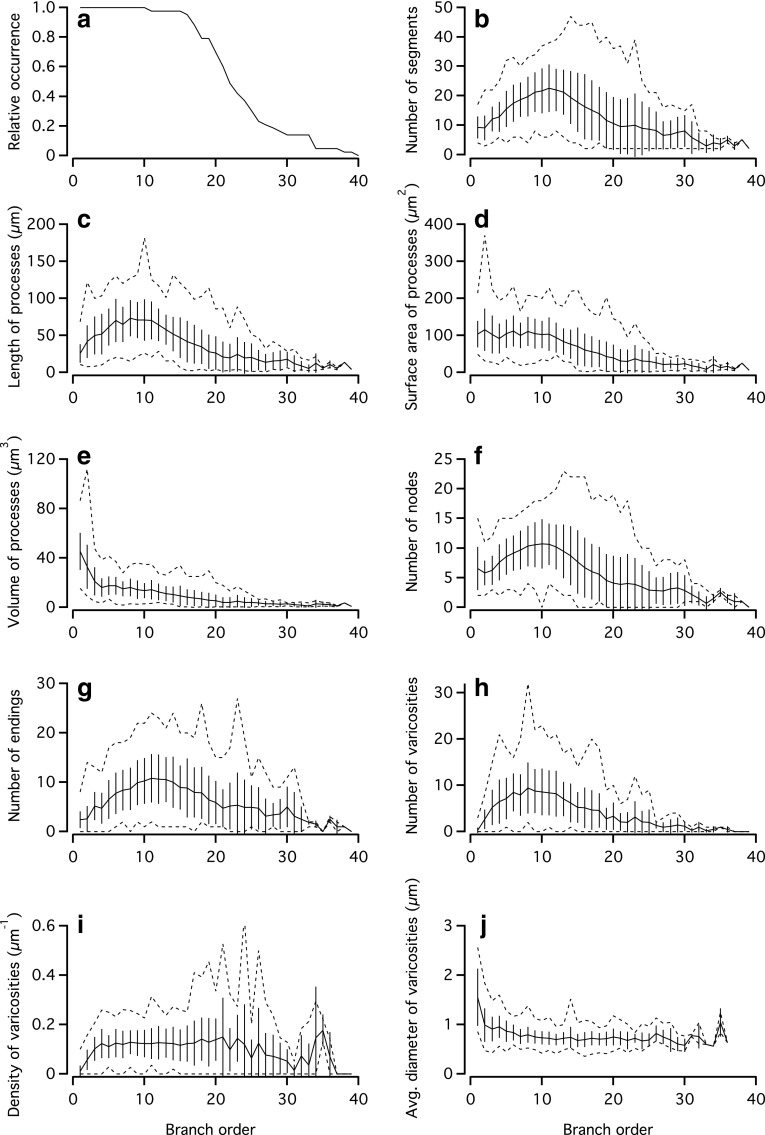
Dendritic parameters as a function of segment branch order for AII amacrine cells (using the central shaft branch ordering scheme). **a** Relative occurrence of dendritic segments of a given branch order for the population of quantitatively reconstructed AII amacrine cells. All cells contained segments with branch order up to and including 10 and the highest branch order observed for any cell was 39. **b**–**j** Different dendritic parameters versus branch order for the same AII amacrine cells as in **a**. For any given branch order, data are plotted as mean (*continuous line*) ± SD (*vertical lines*) and range with maximum and minimum values (*dashed lines*)

Using the central shaft branch ordering scheme, Fig. 8a shows the branch order frequency distribution for all cells reconstructed. All cells contained segments of branch orders from 1 to 10 and ~70 % of the cells contained at least one segment of branch order 20. From branch order 20 to 30 there was a steep decline in the proportion of cells with a given branch order, followed by a more shallow reduction from branch order 30 to 39 (Fig. 8a). The dendritic branching was analyzed as a function of branch order with respect to the number of segments (Fig. 8b), total length of processes (Fig. 8c), total surface area of processes (Fig. 8d), total volume of processes (Fig. 8e), and the number of nodes (Fig. 8f) and endings (Fig. 8g). Except for surface area (Fig. 8d) and volume (Fig. 8e), all these parameters displayed a skewed distribution with a peak close to branch order 10. When the dendritic varicosities were analyzed in the same way, we also observed a skewed distribution for the average number of varicosities for segments of a given branch order, with a peak located at branch order 8 (Fig. 8h). The average density of varicosities for segments of a given branch order displayed minima for the lowest and highest branch orders, with a shallow plateau of approximately 0.10–0.15 varicosities/µm in between (Fig. 8i). The average varicosity diameter displayed a maximum at branch order 1, followed by a gradual reduction with increasing branch order (Fig. 8j). When we performed the same analyses with the centrifugal branch ordering scheme, the results were relatively similar, but with the peak of the corresponding distributions shifted towards higher branch orders (typically between 10 and 20; data not shown).

### Dendritic parameters as a function of location in the IPL

In addition to being postsynaptic to rod bipolar cells at dyad synapses made onto the arboreal dendrites in the inner (proximal) part of the inner plexiform layer, AII amacrine cells are also postsynaptic to (some) OFF-cone bipolar cells at dyad synapses made onto lobular appendages in the outer (distal) part of the inner plexiform layer (Kolb and Famiglietti 1974; Famiglietti and Kolb 1975; Kolb 1979; Strettoi et al. 1992; Singer and Diamond 2003; Tsukamoto and Omi 2013). Whereas the arboreal dendrites have not been found to be presynaptic at chemical synapses, ultrastructural evidence suggests that the lobular appendages can be presynaptic to axon terminals of OFF-cone bipolar cells and dendrites of OFF-ganglion cells (Kolb 1979; Strettoi et al. 1992), supported by recent evidence for voltage-gated Ca^2+^ channels and synaptic output at lobular appendages, but not at arboreal dendrites (Habermann et al. 2003; Balakrishnan et al. 2015).

**Fig. 9:**
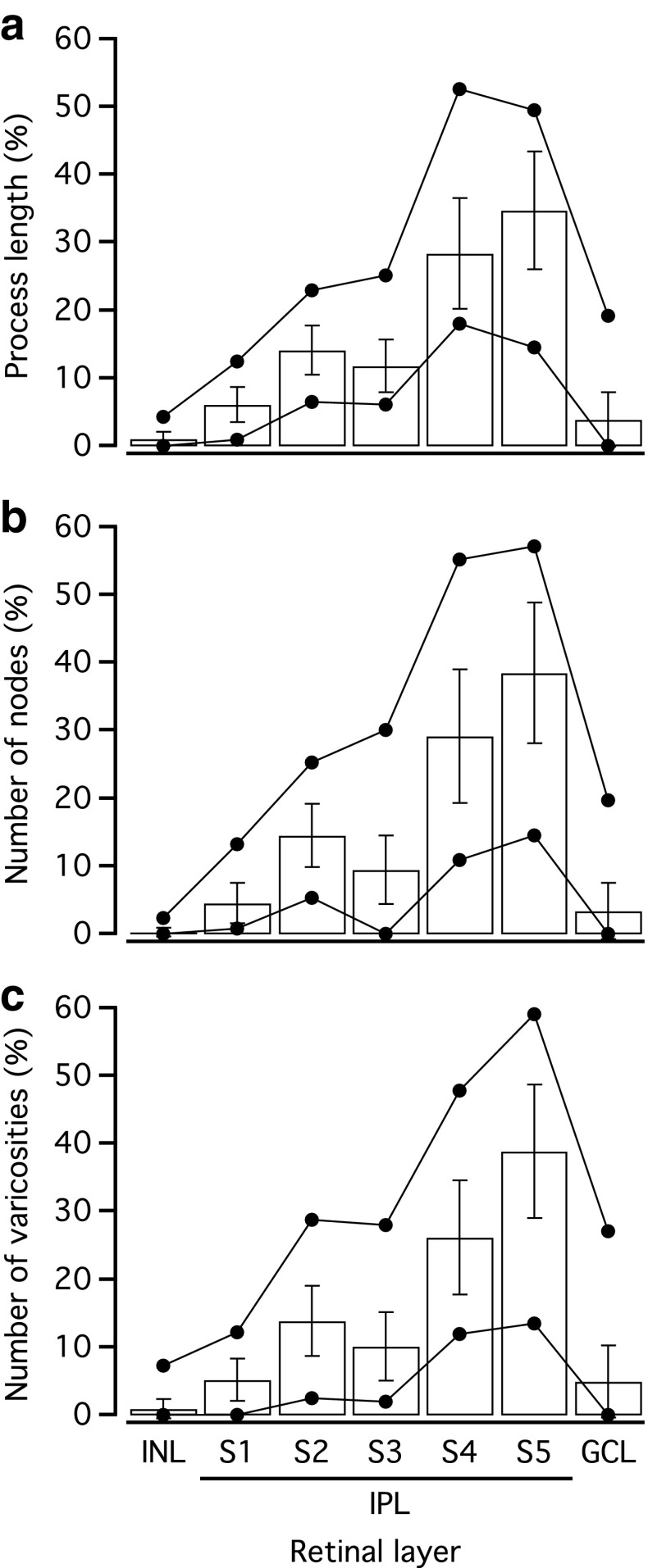
Dendritic parameters as a function of location in retinal layers for AII amacrine cells. **a**–**c** The relative fraction of process length (**a**), number of nodes (**b**), and number of varicosities (**c**) (see “Results” for operational definition of varicosity) as a function of location in retinal layers (INL, IPL, and GCL) and strata of the IPL (S1–S5). Data are plotted as mean ± SD and the maximum and minimum values of the range are indicated by *filled circles* connected by *straight lines*

Given the fact that AII amacrine cells have a bistratified morphology and that the specificity of the synaptic connections is a function of location in the inner plexiform layer, we decided to analyze process length, number of nodes, and number of varicosities in relation to the location across the different strata of the inner plexiform layer. For each cell, the borders of the inner plexiform layer were demarcated by eye on a representative image acquired by IR-LSGC microscopy (performed in parallel with the fluorescence imaging). The inner plexiform layer was then divided into five equally thick strata (S1–S5; Fig. 5). The digital reconstructions were projected directly onto these images and used to estimate the relative proportions of process length (Fig. 9a), number of nodes (Fig. 9b), and number of varicosities (Fig. 9c) in S1–S5. Because some processes can extend into either the inner nuclear layer or the ganglion cell layer (Fig. 5), these layers were also included in the analysis. For all three parameters, there was a clear bistratified distribution with peaks in S2 and in S4–S5 (Fig. 9). The inner nuclear layer and the ganglion cell layer contained a small proportion of all three elements as well, with the fraction in the ganglion cell layer consistently higher than in the inner nuclear layer. The results were very similar when we examined the distributions for the same parameters by using absolute instead of relative values (not shown).

### Dendritic parameters as a function of dendritic field area and retinal eccentricity

**Fig. 10:**
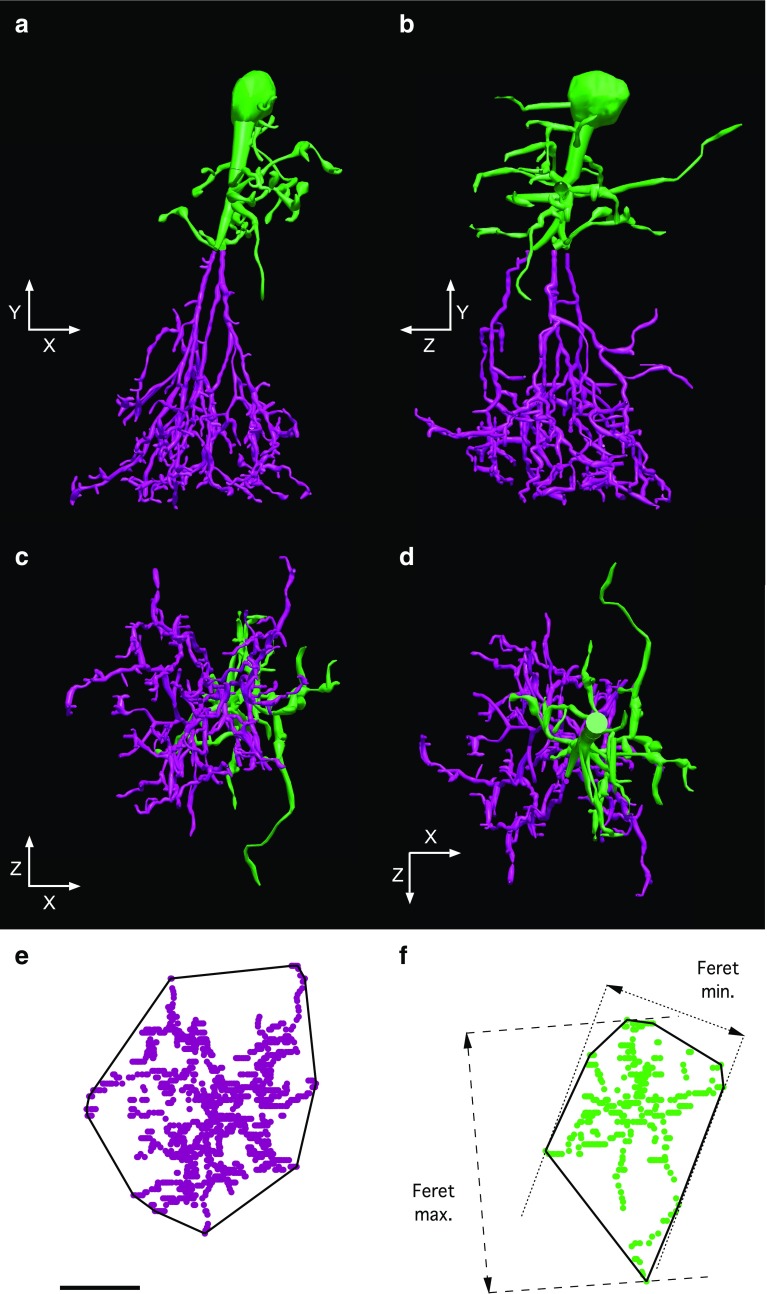
Arboreal (proximal region of the inner plexiform layer) and lobular (distal region of the inner plexiform layer) dendritic fields of AII amacrine cells. **a** 3D view from the front (along the *Z* axis according to the spatial coordinates defined during MPE microscopic imaging) of a morphologically reconstructed AII amacrine cell. The distal (relative to the thickness of the retina) region of the cell corresponding to the cell body, apical dendrite and lobular dendrites is colored in *green* and the proximal region corresponding to the arboreal dendrites is colored in *magenta*. **b** As in **a**, but viewed from the side (along the *X* axis). **c**, **d** As in **a**, but viewed from the *bottom* (**c**) or *top* (**d**) along the *Y* axis after digitally removing the soma. **e** The projection of the arboreal (proximal) dendritic field viewed from the *bottom* (as in **c**) onto the *XZ* plane. Each *dot* corresponds to a reconstruction point. Here and in **f**, the *continuous black line* indicates the 2D convex hull for the corresponding dendritic field. **f** The projection of the lobular (distal) dendritic field viewed from the *bottom* (as in **c**) onto the *XZ* plane. Feret maximum and Feret minimum correspond to the largest and smallest caliper widths of the dendritic field, respectively. *Scale bar* 10 µm (for the dendritic fields in **e**, **f**)

One well-established source of morphological variability between AII amacrine cells is related to the degree of retinal eccentricity. The distribution and spatial density of AII amacrines have been investigated in detail for several mammalian species and it is well established that they depend on the location and eccentricity. In the rat the density increases towards the center and is higher in the superior than in the inferior retina (Wässle et al. 1993). The size of the dendritic fields changes in the opposite direction, i.e. it decreases towards the center. In our study, it was not possible to keep track of the eccentricity (center-to-periphery location) of the cells in the in vitro slices. Instead, we measured the size of the dendritic field (as projected onto the *XZ* plane; corresponding to the retinal surface) and used this property as an indirect measure of retinal eccentricity. Before making the measurements, each cell was rotated in the *XY* and *YZ* planes as required to ensure that the major axis was oriented approximately vertically in both planes (Fig. 10a, b). For each cell we defined two 2D convex hulls, one for the projection of the arboreal dendrites (Fig. 10c–e) and one for the projection of the lobular dendrites (Fig. 10c, d, f). Because arboreal dendrites can arise from processes starting out as lobular dendrites, the two dendritic fields were not defined by classification of processes as such, but instead by separate projections of the parts of the dendritic arborization located in the proximal and distal region of the inner plexiform layer. These were separated by an *XZ* plane, the location of which was set by eye along the *Y* axis (Fig. 10a, b). For the cell illustrated in Fig. 10, the surface areas of the compartments distal (green in Fig. 10a, b) and proximal (purple in Fig. 10a, b) to the *XZ* plane constituted 37 and 63 % of the total surface area (calculated as the sum of the soma surface area and the dendritic surface areas, cf. Table 1), respectively. For the whole population of reconstructed cells (*n* = 43), the corresponding values were 40.9 ± 7.7 % (range 23.0–59.3) and 59.1 ± 7.7 % (range 40.7–77.0).

**Fig. 11:**
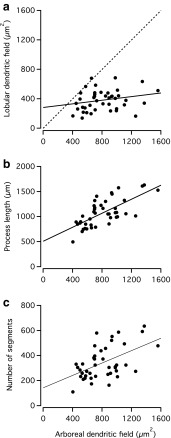
Morphological properties of AII amacrine cells as a function of arboreal dendritic field area. **a** Lobular dendritic field area versus arboreal dendritic field area. Here and in **b** and **c**, each data point corresponds to an individual cell. A *straight line* (*continuous*) has been fitted to the data points. The *dashed line* corresponds to the identity line, i.e. identical area of arboreal and lobular dendritic fields. Notice that only for one cell is the area of the lobular dendritic field larger than that of the arboreal dendritic field. **b** Total length of all processes versus arboreal dendritic field area. A *straight line* has been fitted to the data points. **c** Total number of branch segments versus arboreal dendritic field area. A *straight line* has been fitted to the data points

For each 2D convex hull we calculated area, perimeter and Feret maximum and minimum (Fig. 10e, f). As displayed in Table 1, the average area of the 2D convex hull for the arboreal dendritic fields was 810 µm^2^, with a range from 400 to 1560 µm^2^. For the lobular dendritic fields the average was 380 µm^2^, with a range from 140 to 690 µm^2^. There was a weak positive correlation between the areas of the arboreal and lobular dendritic fields and for all except one cell, the area of the arboreal dendritic field was larger than that of the lobular dendritic field (Fig. 11a).

The difference in dendritic field size between AII amacrine cells at different eccentricities could correspond to a simple scaling of cell size or, in contrast, it could correspond to a difference in branching complexity. To examine this, we first plotted total process length as a function of arboreal dendritic field area (Fig. 11b). There was an almost linear relationship between these two parameters, indicating that a larger dendritic field size is not simply generated by a different structural organization of a constant total length of dendritic processes. To investigate whether the increase in dendritic field size and total process length primarily corresponds to a simple scaling or rather to an increase in branching complexity, we plotted the number of dendritic segments (equal to the sum of the number of nodes and the number of endings) as a function of the arboreal dendritic field area (Fig. 11c). There was a clear positive correlation between arboreal dendritic field area and the number of segments, such that the AII amacrine with the smallest area (400 µm^2^) had 110 segments and the AII amacrine with the largest area (1560 µm^2^) had 478 segments. This strongly suggests that AII amacrines at different eccentricities are not simply scaled versions of each other. For all AII amacrine cells, the number of nodes was very similar to the number of endings, reflecting the overall small number of dendritic trees for each cell. For a single bifurcating dendritic tree, the number of endings equals the number of nodes plus one. Our numbers show a small deviation from this relation (Table 1), most likely due to spurious nodes (accidentally generated or left behind during reconstruction) and/or trifurcations in the reconstructed trees.

As expected, similar to process length, surface area and volume were also positively correlated with the arboreal dendritic area (not shown). Other properties such as maximum branch order, cell body Feret maximum and projection area (in the *XY* plane), and number of dendritic trees displayed no or only minimal correlation with the arboreal dendritic field area (not shown). For the arboreal dendritic fields, increasing area corresponded to an increase of both the Feret maximum and the Feret minimum, as well as an increase of the 3D convex hull volume (calculated from the complete branching of all dendritic trees for each cell; not shown).

### Relationship between arbor volume and branch density

**Fig. 12:**
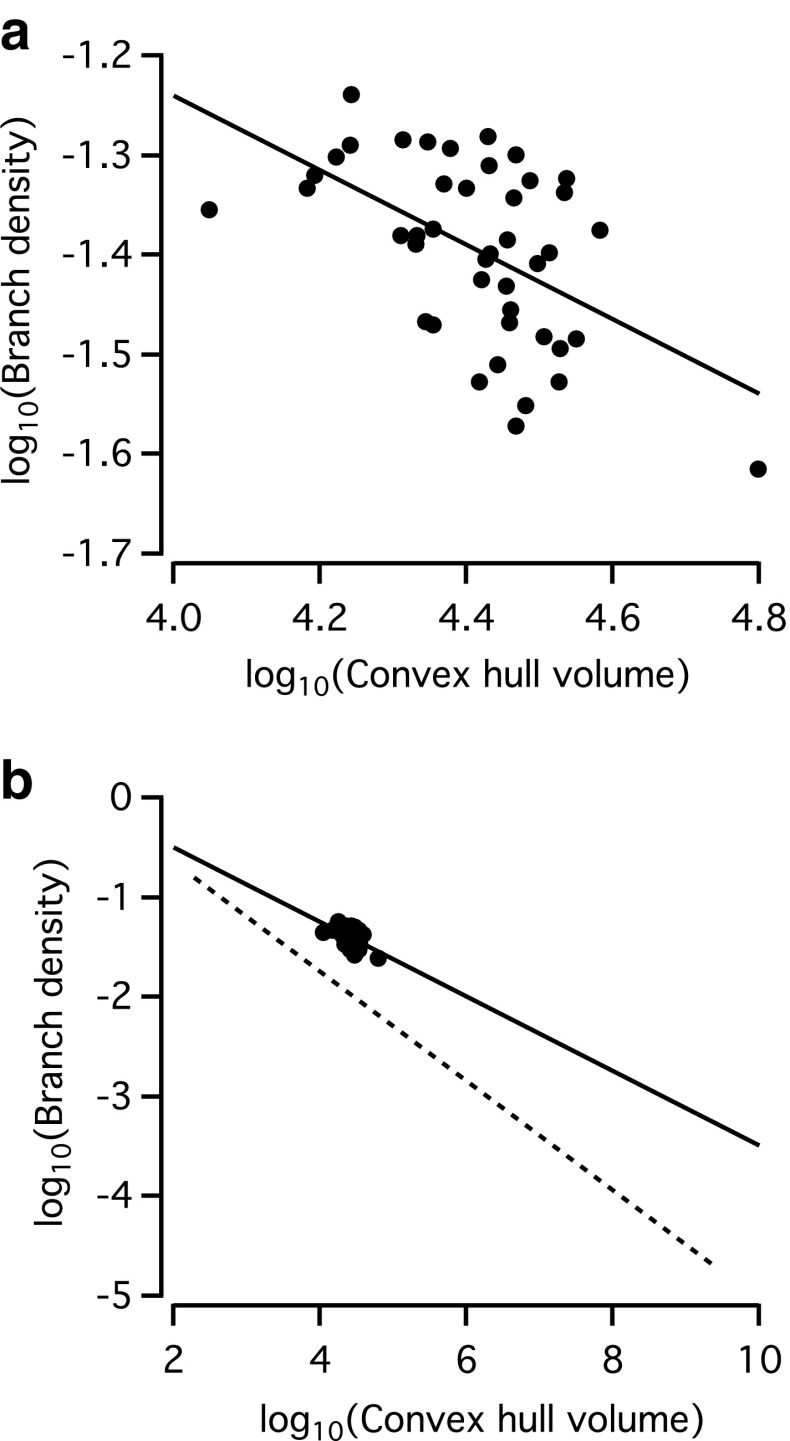
The relationship between the arbor volume and branch density of AII amacrine cells. **a** Branch density (defined as total dendritic branch length divided by the convex hull volume; in µm^−2^) versus convex hull volume (in µm^3^) of the dendritic trees. Each data point corresponds to an individual cell. A *straight line* has been fitted to the data points and has a slope of −0.37 and an intercept of 0.26. **b** The same data points as in a, replotted at the axes range illustrated for a larger population of different neurons (pyramidal neurons and interneurons from rat, cat, monkey, and human) analyzed by Teeter and Stevens (2011). The *continuous line* is the same fitted line as in **a** extrapolated over the larger range. The *dashed line* corresponds to the line fitted to the data points of Teeter and Stevens (2011), with a slope of −0.55 and an intercept of 0.45

Teeter and Stevens (2011) recently reported a general structural design (scaling) principle of neural arbors with a systematic decrease in arbor density with an increase in territory size. As demonstrated by the increased number of nodes, endings and segments with increasing arboreal dendritic field size, as well as the positive correlation between arboreal dendritic field size and 3D convex hull volume (see “Dendritic parameters as a function of dendritic field area and retinal eccentricity”), AII amacrine cells with larger dendritic trees display more complex branching. From these results, however, it is not clear how the density of branching increases with an increase in territory size. To analyze this, we calculated the average branch density as the total dendritic branch length divided by the territory volume, with the latter defined by the volume of the 3D convex hull. As shown in Fig. 12a, there was an inverse relationship between the 3D convex hull volume and the branch density. We analyzed the relationship in logarithmic space and fitted the relationship with a straight line. The slope of the fitted line between the volume and the density in logarithmic space was −0.37 and corresponds to an exponent in linear space (cf. Teeter and Stevens 2011). Even though the data points for AII amacrines cover a much smaller range than the total population of cells analyzed by Teeter and Stevens (2011), it is clear that AII amacrine cells adhere to the general design principle they discovered, with a decrease in branching density for an increase in arbor territory. AII amacrine cells are small neurons, and their convex hull volume is in the lower range of the neurons analyzed by Teeter and Stevens, but on average AII amacrines display a relatively higher branching density (Fig. 12b).

### Sholl analysis reflects the bistratified morphology

Sholl analysis (Sholl 1953) has been used in numerous studies to characterize and compare the morphological characteristics of single neurons and provides easily interpretable metrics of complex neuronal arborizations (Capowski 1989). For the AII amacrine cells, we performed Sholl analysis by using Neurolucida Explorer to generate a set of nested, concentric spheres (1 µm separation) centered at the centroid of the cell body and obtain a series of morphological parameters as a function of distance from the cell body, calculated either as a crossing with a specific sphere or contained in the shell between two neighboring spheres.

**Fig. 13:**
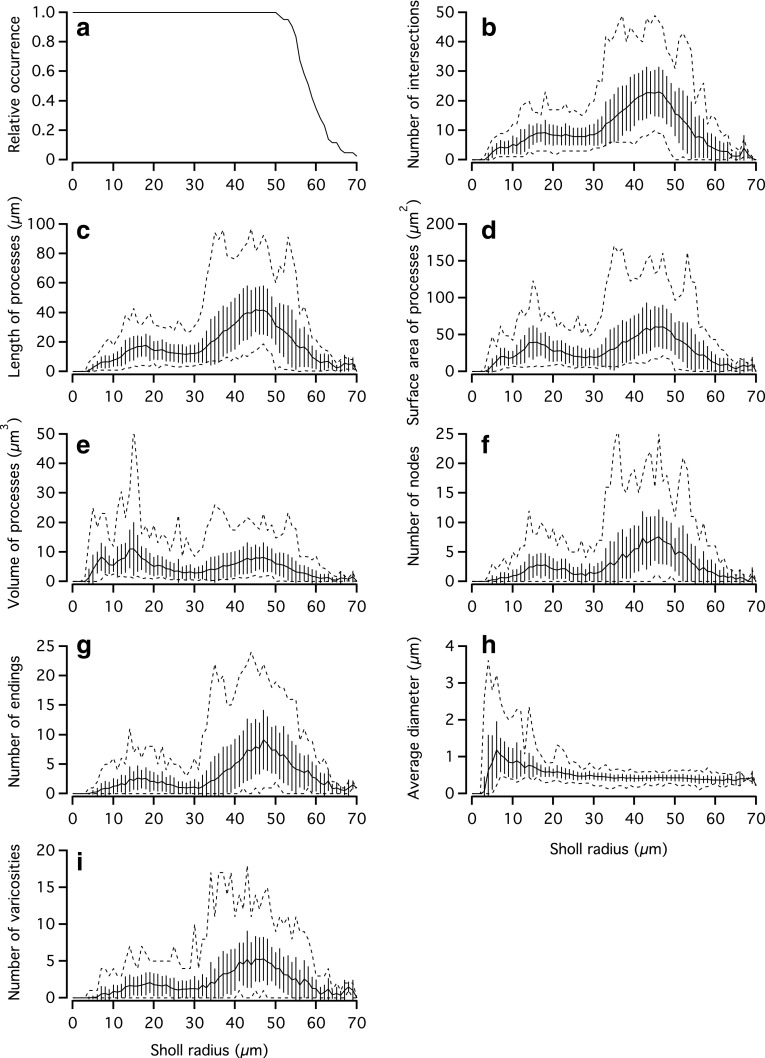
Sholl analysis of AII amacrine cells. **a**–**i** A set of nested concentric spheres (1 µm separation) were centered at the centroid of the cell body and a number of morphological parameters were counted as a function of distance, either as a crossing with a specific sphere or contained in the shell between two neighboring spheres. **a** Relative occurrence of Sholl spheres crossed by processes at a given distance from the cell body centroid. All cells crossed spheres with radii up to and including 50 µm and the largest sphere had a radius of 69 µm. **b**–**i** Different morphological parameters as a function of Sholl sphere radius. Data are plotted as mean (*continuous line*) ± SD (*vertical lines*) and the maximum and minimum values of the range are indicated (*dashed lines*). The distribution of all properties (except average dendritic diameter; **h**) reflects the bistratified morphology of AII amacrine cells

For the 43 cells, the average radius of the outermost sphere was 58 ± 4.4 (range 50–69 µm). Figure 13a shows that all cells reconstructed contained Sholl spheres with radii ≤50 µm, corresponding approximately to the thickness of the inner plexiform layer. The relative occurrence dropped sharply for Sholl radius values >50 µm and at 60 µm the relative occurrence was only 0.35 (Fig. 13a).

Figure 13b shows the number of intersections (crossings) between dendritic processes and spheres as a function of distance from the cell body. The largest number of crossings occurred at a radius of approximately 45 µm, dominated by S4 and S5 of the inner plexiform layer. In addition, there was a tendency to a bimodal distribution with a smaller peak at a radius of approximately 18 µm, dominated by S2 of the inner plexiform layer.

When we analyzed length, surface area and volume of processes in the same way, there was a clear bimodal distribution (Fig. 13c–e), with variable relative magnitude of the two peaks. Irrespective of the relative magnitude of the two peaks, they were located approximately at 15–20 µm (dominated by S2) and at 45 µm (dominated by S4–S5). We also analyzed the number of nodes and the number of endings in the same way. As illustrated in Fig. 13f, g, there was a pronounced bimodal distribution, with peak locations similar to the other parameters and the larger peak around 45 µm (dominated by S4–S5). Average process diameter did not display a bimodal distribution, but reached a peak of approximately 1 µm at a Sholl radius of 6 µm (in S1), close to the cell body, and then decayed to a plateau of approximately 0.4 µm (Fig. 13h). As discussed earlier, because of the resolution limit of light microscopy, there is considerable uncertainty with respect to the true diameters of the thinnest reconstructed processes of AII amacrine cells. Finally, the number of varicosities (see “Quantitative analysis of dendritic varicosities”) also displayed a bimodal distribution, with peaks around 20 and 45 µm (Fig. 13i).

### Quantitative analysis of dendritic varicosities

**Fig. 14:**
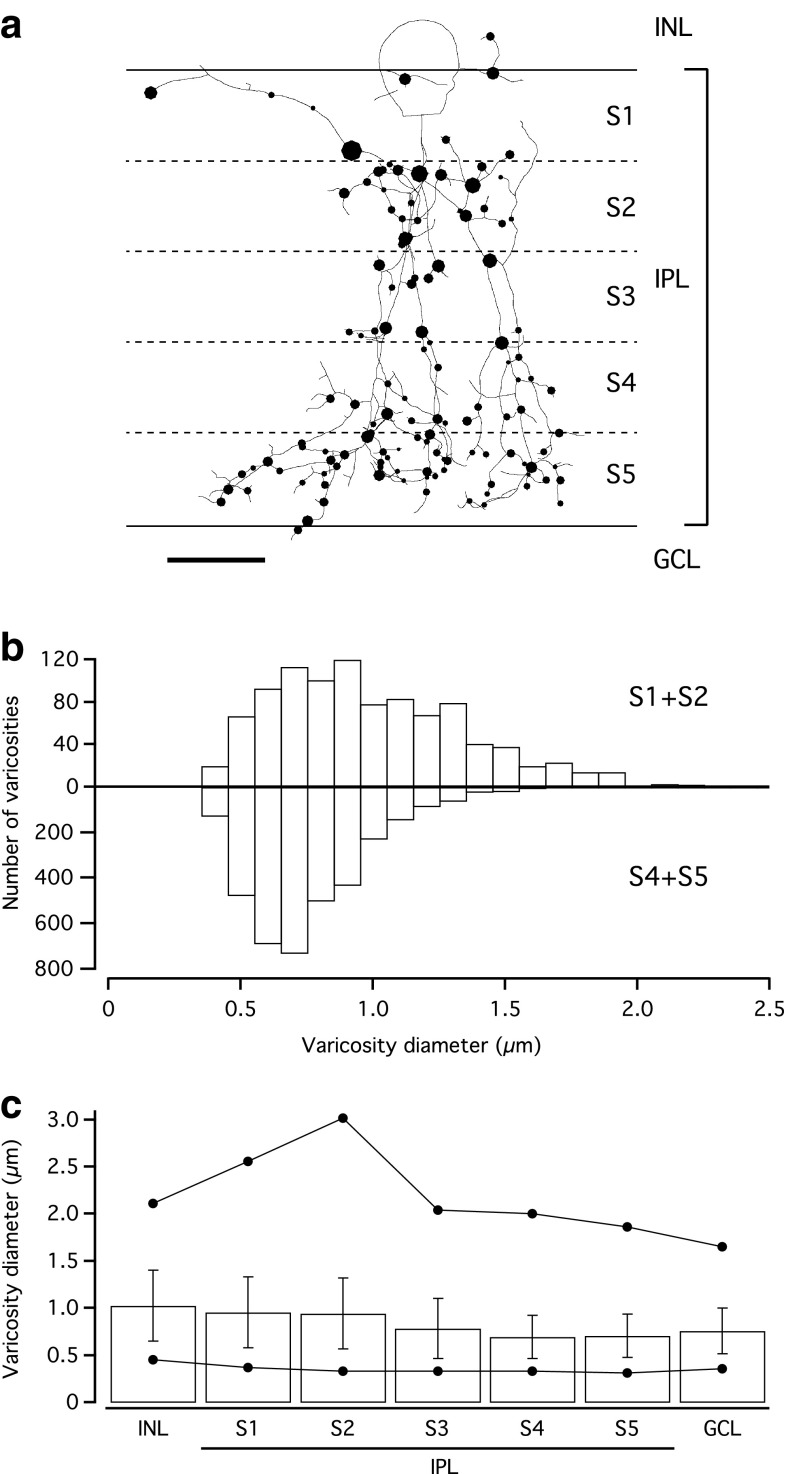
Size and laminar distribution of dendritic varicosities of AII amacrine cells. **a** Example of size and distribution of dendritic varicosities of an AII amacrine cell. The dendritic tree of the reconstructed AII is displayed as a skeleton structure with no indication of the diameter of the different processes and the cell body is displayed by the largest contour. A varicosity was defined as a spatially discrete swelling where the maximum diameter increased ≥80 % relative to the diameter immediately before and after the swelling. Each varicosity is marked by a *filled sphere* with diameter and location corresponding to the largest *circle* that would fit inside the varicosity. Retinal layers and strata as in Fig. 5. **b** Frequency distribution of the diameters of AII amacrine varicosities located in S1 + S2 and in S4 + S5 (for all cells). Notice that whereas there are many more varicosities in S4 + S5, the distribution is shifted towards larger diameters for varicosities in S1 + S2. **c** Varicosity diameter as a function of location in retinal layers (INL, IPL, and GCL) and S1–S5 of the IPL (for all cells). Data are plotted as mean ± SD and the maximum and minimum values of the range are indicated by *filled circles* connected by *straight lines*. Notice that the largest maxima are found in S1 and S2 and that the average diameters in S1 and S2 are approximately 35 % larger than those in S3–S5. *Scale bar* 10 µm (**a**)

The most pronounced and well-characterized dendritic swellings or varicosities of AII amacrine cells are located along and at the ends of the lobular dendrites (Figs. 4, 5). In addition, however, there are a number of distinct swellings along the large majority of arboreal dendrites (Figs. 4, 5). In an ultrastructural analysis of AII amacrine cells in cat retina, Sasaki-Sherrington et al. (1984) found that each varicosity contained at least one mitochondrion or a “smooth vesicular body” and the presence of either organelle produced a varicosity. A large proportion of varicosities (~80 %) corresponded to sites of synaptic inputs (Sasaki-Sherrington et al. 1984). On this background, we decided to investigate quantitatively the localization and distribution of varicosities in our reconstructed AII amacrine cells. For our analysis, a dendritic varicosity was operationally defined as a spatially discrete swelling where the maximum diameter increased ≥80 % relative to the diameter immediately before and after the swelling. Figure 14a illustrates the location of all detected varicosities for an AII amacrine cell (with the neuronal tree displayed as a skeleton) and each varicosity has been marked by a *sphere* with diameter corresponding to the size of the varicosity (as described above). For this cell, we detected a total of 128 varicosities with an average diameter of 0.85 ± 0.30 µm (range 0.39–2.10 µm). The average distance to the nearest neighbor was 2.7 ± 2.2 µm (range 0.6–7.7 µm). When the same analysis was performed for all 43 AII amacrines, the number of varicosities was 125 ± 40 (range 59–268), with an average diameter of 0.77 ± 0.10 µm (range 0.39–2.7; *n* = 5375 varicosities). When we averaged the diameters for each cell, the range for all the averages was 0.57–1.0 µm. The average distance to the nearest neighbor was 3.0 ± 0.4 µm (range 2.2–4.0), with an average closest distance of 0.91 ± 0.22 µm (range 0.54–1.52) and an average farthest distance of 9.7 ± 3.0 µm (range 5.6–17.5).

The laminar analyses illustrated in Fig. 9c indicated that the density of varicosities displayed a bistratified organization, with peaks in S2 and S4–S5, but that the number of varicosities is considerably larger in S4–S5 than in S2. Nevertheless, the distribution of varicosity diameters in S1–S2 was skewed towards larger values compared with that for varicosities in S4–S5 (Fig. 14b), corresponding to the presence of the lobular appendages in S1 and S2. Because of the relatively large number of smaller varicosities in all strata of the inner plexiform layer, the average varicosity diameter does not vary much between the different layers and strata, but the maximum diameter is clearly higher in S1 and S2 than in any other strata or layers (Fig. 14c).

### Principal component and cluster analysis reveals morphological homogeneity of reconstructed cells

Generally speaking, PCA decomposes a dataset into a set of perpendicular vectors (principal components) and sorts the vectors according to their eigenvalues (i.e., according to how much of the variance in the data each vector explains). The first few principal components typically represent a genuine influence of underlying variables, while the later ones typically represent random fluctuations. Our data showed two principal components with eigenvalues above chance level (Table 2). Together these two principal components explain over 40 % of the variance in the morphometry data. In Table 2, values with *z* scores >2 are highlighted in bold, corresponding to *p* < 0.05 when not correcting for multiple comparisons.

**Table 2:** Principle components of the cells’ metrics

	PC 1	PC 2
Eigenvalue	6.9	4.1
Fraction of total variability accounted for by principal component	26 %	15 %
Coefficients		
Soma surface area (µm^2^)	−4.9	**24**
Soma projection Feret maximum (µm)	−0.0016	**0.49**
Number of primary dendrites	0.062	0.18
Length of main primary dendrite (µm)	0.040	−0.77
Maximum diameter of main primary dendrite (µm)	−0.024	0.063
Dendritic length of inner nuclear layer (µm)	**2.1**	1.5
Dendritic length of S1 (µm)	**4.5**	3.6
Dendritic length of S2 (µm)	**11**	2.4
Dendritic length of S3 (µm)	**8.4**	**9.8**
Dendritic length of S4 (µm)	**40**	16
Dendritic length of S5 (µm)	**26**	−**29**
Dendritic length of ganglion cell layer (µm)	**5.6**	−**9.1**
Dendritic surface area (µm^2^)	**200**	−68
Average dendritic diameter (µm)	0.0085	−**0.018**
Average branch segment path length (µm)	−**0.11**	**0.13**
Maximum branch order (central shaft ordering)	**1.9**	−0.52
Average partition asymmetry	**0.0071**	−0.0065
Number of nodes	**22**	−7.7
2D convex hull Feret max., arboreal dendrites (µm)	**1.6**	0.66
2D convex hull Feret min., arboreal dendrites (µm)	**1.3**	**0.83**
2D convex hull Feret max., lobular dendrites (µm)	**1.2**	**1.9**
2D convex hull Feret min., lobular dendrites (µm)	0.54	**0.92**
3D convex hull volume, dendritic tree (µm^3^)	**2400**	**2000**
Euclidean distance from soma (mean) (µm)	0.063	0.26
Euclidean distance from soma (maximum) (µm)	0.29	**1.0**
Contraction	−**0.0026**	0.0014
Fractal dimension	0.0008	−0.0015
Regression coefficients of excluded metrics		
Soma volume (µm^3^)	−5.9	**40**
Dendritic length (µm)	**97**	−4.6
Dendritic volume (µm^3^)	**32**	−11
Maximum branch order (centrifugal ordering)	**2.0**	−1.0
Number of nodes in S4	**9.2**	1.0
Number of nodes in S5	**6.7**	−7.0
Number of nodes in IPL	**20**	−6.1
Number of endings	**22**	−6.7
2D convex hull area, arboreal dendrites (µm^2^)	**71**	33
2D convex hull area, lobular dendrites (µm^2^)	24	**40**
3D convex hull surface area, dendritic tree (µm^2^)	**260**	250

Only PC 1 and PC 2 were found to be statistically significant. Individual components with *z* scores >2 (see “Materials and methods”) are highlighted in bold. Regression coefficients (with PC 1 and PC 2) of excluded metrics are tabulated for metrics that showed correlation with at least one of the two PCs (*R* ^2^ > 0.4; highlighted in bold)

The first principal component has large coefficients for the area and volume spanned by the cell and its dendritic length. Its direction reflects how the morphological properties of AII amacrine cells scale with their size. For example, a cell is expected to scale such that when its spanned volume is about 2400 µm^3^ above average, it has 22 more nodes (branch points), 11 µm longer dendritic length in S2 and 0.11 µm less average distance between branch points (Table 2, PC1). The second principal component suggests that there is a second design principle that underlies the cells’ morphologies. It shows that for cells that have approximately the same number of nodes and total dendritic length (excluded metrics; cf. Table 2), spanning a larger area and volume is correlated with having relatively less dendritic length in S5 and in the GCL, as well as having a larger soma (Table 2, PC2).

**Fig. 15:**
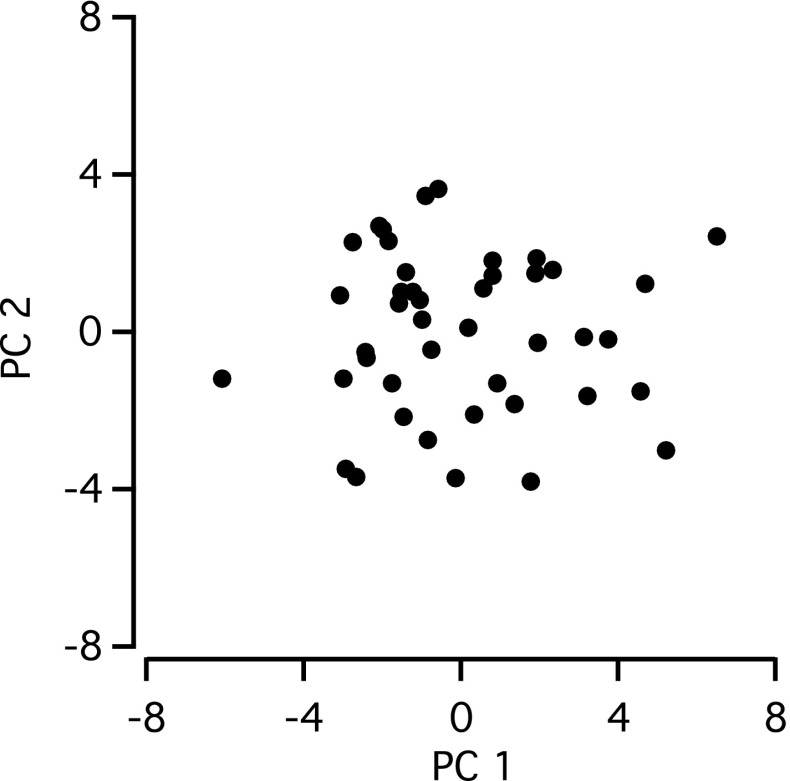
Distribution of the first two principal components (PC 1 and PC 2) obtained by principal component analysis of the morphological parameters of AII amacrine cells. PC 1 and PC 2 were the two principal components with eigenvalues above chance level and the scatter plot shows each cell’s projection onto PC 1 and PC 2. Notice that there is no evidence for separable clusters

Cluster analysis (see “Materials and methods”) revealed no separable clusters in our data, as is also evident from the scatter plot of each cell’s projection onto the first two principal components (Fig. 15). This is consistent with previous evidence that AII amacrine cells constitute a unique population of cells and strongly suggests that all cells reconstructed and included in the analysis are genuine AII amacrine cells.

### Morphology of AII amacrine cells visualized by biocytin histology

An alternative to obtaining the cellular morphology by imaging fluorescence after filling cells with dyes during electrophysiological recording is to fill them with tracers like biocytin and Neurobiotin. These tracers cannot be visualized during recording, but must be visualized following the binding to streptavidin (or avidin) which can be linked to either a fluorescent dye (for visualization by fluorescence microscopy) or HRP (for visualization after developing an insoluble reaction product). In a previous study from our laboratory (Veruki et al. 2010), we filled AII amacrines in rat retinal slices with biocytin from patch pipettes and reconstructed the morphology after histochemical detection. Because AII amacrines are connected to other AIIs and ON-cone bipolar cells via gap junctions (Kolb and Famiglietti 1974; Famiglietti and Kolb 1975; Kolb 1979; Strettoi et al. 1992; Chun et al. 1993) and because biocytin and Neurobiotin can diffuse into neighboring cells through gap junctions (Vaney 1991), we limited the recording time to 5–10 min. Because we were interested in comparing this workflow with that established in the current study, we have re-analyzed the digital reconstructions obtained by biocytin histology for seven AII amacrine cells (see Veruki et al. 2010). The primary goal was to find out if the relatively brief time allowed for intracellular diffusion of biocytin resulted in reconstruction of neuronal trees similar in size and branching complexity to those obtained with MPE microscopy. With respect to the number of primary dendrites, there was no difference between the dye-filled (3.4 ± 1.9; Table 1) and the biocytin-filled cells (3.7 ± 1.5, range 2–6, *p* = 0.63, *t* test). However, the number of endings was approximately five times larger for the dye-filled cells (178 ± 66; Table 1) than for the biocytin-filled cells (34 ± 8, range 26–48, *p* ≪ 10^−6^, *t* test) and dendritic length was approximately three times larger for the dye-filled cells (1080 ± 270 µm; Table 1) than for the biocytin-filled cells (359 ± 68 µm, range 247–450, *p* ≪ 10^−6^, *t* test). Although we have not compared morphological reconstructions after filling the same cells with both biocytin and a fluorescent dye, these results suggest that for AII amacrine cells, biocytin histology is suboptimal when the goal is to obtain complete cellular morphologies without risk of filling processes of neighboring coupled cells.

In the previous study with reconstructions obtained by biocytin histology (Veruki et al. 2010), the expected capacitance, calculated by multiplying the total surface area with the standard value of 0.01 pF/µm^2^ for specific membrane capacitance (Major 2001), was lower than the capacitance automatically estimated by the neutralization circuitry of the patch clamp amplifier. This was interpreted as suggesting that the automatic measurements were influenced by the capacitance of other cells, electrically coupled via gap junctions to the cell from which the recording was made. In the present study, however, the expected capacitance (calculated by multiplying the total surface area with the standard value for specific membrane capacitance) was 20.9 ± 5.7 pF (range 12.6–32.9; *n* = 30 cells recorded with CSEVC amplifiers), larger than the capacitance estimated by the neutralization circuitry (14.7 ± 3.7 pF; range 6.1–19.9; *p* < 10^−4^, paired *t* test). The larger surface area and expected capacitance of the cells in the present study compared to the cells in the previous study (Veruki et al. 2010), are most likely explained by the incomplete cellular morphology obtained with biocytin histology. The difference between the expected and automatically measured capacitance in the present study could suggest that the specific membrane capacitance of AII amacrine cells is lower than 0.01 pF/µm^2^, but could also be related to inaccurate estimates of capacitance by the neutralization circuitry. In effect, this circuitry attempts to fit the decay of capacitative currents evoked by square-wave pulses with a single-exponential function. For branched neurons like AII amacrine cells, the decay cannot be satisfactorily described by a single-exponential function and a more robust analysis will require compartmental modeling with morphological reconstruction and electrophysiological recording from the same neurons (e.g. Oltedal et al. 2009).

## Discussion

In this study we have used MPE microscopy to acquire image stacks of dye-filled AII amacrine cells in live retinal slices. Using post-acquisition deconvolution, we increased the spatial resolution of the image stacks that were subsequently used for detailed and accurate quantitative digital reconstruction of neuronal morphologies. We then performed morphometric analysis of these digital reconstructions and extracted pertinent quantitative information, encompassing distribution statistics, e.g. the number of nodes and branch segments as a function of the distance from the soma, as well as geometric, size-related and topological properties of the cells (see Capowski 1989 for an overview). In the following, we will discuss the most important results, how our study compares with earlier studies of AII amacrine cells and how our results may facilitate future studies of these cells and the retinal circuits in which they take part.

### Quantitative morphological analysis of AII amacrine cells

Perhaps the most important single result to appear from our study is the unexpected extent of branching of the dendritic tree of AII amacrine cells. There are, admittedly, few studies that have analyzed the extent of neuronal branching quantitatively, but the maximum branch order was considerably higher than previously suggested for AII amacrine cells (in cat retina; Sterling 1983). It is possible that early electron microscopic reconstructions of AII amacrine cells missed a fair number of processes (e.g. Sterling 1983; Sasaki-Sherrington et al. 1984; Sterling et al. 1988; Vardi and Smith 1996) with the main focus being less on complete reconstruction as opposed to identifying synaptic contacts with different types of neurons. However, even images acquired by light microscopy often give the overall impression of more sparse branching than we observed in our study with MPE microscopy. This pertains equally well to images based on wide-field fluorescence microscopy (Vaney 1985; Voigt and Wässle 1987; Boos et al. 1993; Veruki and Hartveit 2002a) and on Golgi impregnation (Famiglietti and Kolb 1975; Perry and Walker 1980; Dacheux and Raviola 1986; Strettoi et al. 1992; Wässle et al. 1995). For wide-field fluorescence microscopy, this is not surprising, as only incomplete image stacks were likely to have been acquired and blurring from out-of-focus fluorescence light degraded the resolution. For Golgi stained material, it cannot be excluded that some branches were missed by the impregnation procedure, but when drawings were generated from the microscope, the goal might well have been to create images that were faithful reproductions of the overall morphology without necessarily capturing every branch. Photographic reproductions of Golgi material have typically only been used to illustrate images at a few focal planes at best. In some studies, intracellular injection of the fluorescent dye Lucifer yellow was followed by photoconversion to an insoluble reaction product (Mills and Massey 1991; Vaney et al. 1991). In more recent studies, dye-filled AII amacrines have been imaged by confocal microscopy (e.g. Meyer et al. 2014). In both cases, the resulting branching pattern seems to be more similar to that obtained in our study, but the quantitative data required for a direct comparison are not available. Recently, presumed complete reconstructions obtained by electron microscopy have been published for AII amacrine cells both from mouse (Tsukamoto and Omi 2013) and rabbit retina (Marc et al. 2014). Although the number of ultrastructurally reconstructed AII amacrine cells is limited, compared to our light microscopic material, the extent of branching seems more similar to that in our study.

The observed extent and variability of branching of the dendritic tree of AII amacrine cells is likely to influence the electrotonic properties of these cells. It has previously been suggested that AII amacrine cells (in cat retina) can be modeled as isopotential cells (Vardi and Smith 1996). The two AII amacrine cells reconstructed by Vardi and Smith (1996) from electron microscopy displayed considerably less branching than the cells in our study. The extent to which the degree of isopotentiality will be influenced by the degree of branching is unknown and a more detailed analysis will require development of compartmental models based on combined electrophysiological and morphological data. In addition, it is important to keep in mind that the extent to which a neuron is isopotential depends on the frequency of the stimulus.

In an earlier study from our laboratory, AII amacrine cells were filled with biocytin during whole-cell recording in live retina slices and the cells were morphologically reconstructed by bright-field microscopy after developing a reaction product (Veruki et al. 2010). These cells displayed less extensive branching and shorter dendritic lengths compared to dye-filled cells imaged by MPE microscopy, most likely because insufficient time was allowed for intracellular diffusion of biocytin to obtain complete visualization. However, because AII amacrine cells are coupled to each other and to ON-cone bipolar cells via gap junctions, it is a problem to use tracers like biocytin and Neurobiotin for single-cell visualization because they can diffuse into the network of coupled cells. In a recent study of visual cortical neurons (Blackman et al. 2014) it was found that with respect to representing the overall morphology, the results obtained by biocytin histology and MPE imaging were very similar when the same cells were filled with both biocytin and a fluorescent dye during whole-cell recording. It was consistently found, however, that reconstructions based on biocytin histology facilitated tracing of more distal collaterals. It should be noted, however, that the fluorescent images used by Blackman et al. (2014) were not processed with postacquisition deconvolution before reconstruction.

The animals used in our study were between 4 and 7 weeks postnatal. Although we are not aware of any studies that have addressed the morphological development of AII amacrine cells, it is generally assumed that the major developmental processes are over by this age, supported by recent physiological evidence suggesting that the output synapses at the lobular appendages are mature at about postnatal day 25 (Balakrishnan et al. 2015). It could be speculated that AII amacrine cells pass through a developmental stage with more profuse branching that is reduced during a subsequent period of “pruning”. Accordingly, we believe that the age of the animals used in our study is sufficient such that the extensive branching reflects the characteristics of mature AII amacrine cells in the mature retina.

### Morphological reconstruction for compartmental modeling

For development of compartmental models used in studies of neuronal computation and signaling, detailed and accurate morphological reconstructions can be a crucial element and should ideally be generated from the same neurons from which electrophysiological data are obtained (Major 2001; Carnevale and Hines 2006). Prior to our study, no such detailed reconstructions have been published and none seem available in any of the publicly accessible databases of morphologically reconstructed neurons (e.g. NeuroMorpho.Org; Ascoli et al. 2007). It was an explicit goal of our study to establish a workflow that can be used for developing compartmental models of AII amacrine cells. Importantly, we imaged cells in live retinal slices filled with fluorescent dyes by diffusion from patch pipettes during whole-cell recording. Because the imaging was performed in parallel with the recording, potential artifacts associated with fixation and processing of the tissue were avoided. Tissue shrinkage during fixation and histological staining procedures is considered a major limitation in cell reconstructions (see Jaeger 2001; Groh and Krieger 2011). An additional reason for obtaining the morphology of live AII amacrine cells with fluorescence imaging instead of biocytin histology is that this tracer can diffuse between cells coupled via gap junctions (Vaney 1991), as is the case for AII amacrines coupled to each other and ON-cone bipolar cells. Because fluorescent dyes like Alexa Fluor 488 and 594 do not diffuse across these gap junctions, they seem ideal candidates to be used for imaging and morphological reconstruction to develop compartmental models.

When wide-field fluorescence imaging was the only fluorescence alternative to light microscopic imaging and reconstruction of biocytin-filled cells, it is clear that biocytin was the preferred alternative (Marx et al. 2012). Wide-field fluorescence imaging suffers from problems related to bleaching and phototoxicity, low contrast and low spatial resolution because of blurring by out-of-focus light. All of this changed dramatically with the introduction of confocal and MPE laser scanning microscopy, with MPE imaging having the added advantage that it can be easily combined with simultaneous electrophysiological recording because of lower phototoxicity. With MPE microscopic imaging and electrophysiological recording performed simultaneously, there is also no need to remove the recording pipette. After a certain recording period, it is often difficult to remove the pipette by pulling an outside-out patch and instead the whole cell body is removed. Especially for small cells where the cell body constitutes a relatively large part of the neuron, this can be a problem. However, it is a disadvantage that MPE microscopy cannot easily be combined with subsequent ultrastructural examination of the tissue. On the other hand, compared to biocytin labeling, MPE microscopic imaging allows the use of different fluorochromes when recording from more than one neuron, thereby making it possible to discriminate between the processes belonging to different cells at points of close contact that potentially correspond to chemical or electrical synapses.

### Variability of morphological properties of AII amacrine cells

There are well-documented differences in the size of dendritic fields (lobular or distal versus arboreal or proximal) as a function of retinal eccentricity, as studied in the retinas of both cat (Vaney 1985), rabbit (Mills and Massey 1991; Vaney et al. 1991; Casini et al. 1995), rat (Wässle et al. 1993), and primate (Wässle et al. 1995). Despite species differences in the extent of regional specialization, the dendritic field sizes increase towards the periphery, concomitant with a decrease in cell density.

In general, the morphological variability within a specific cell type can be puzzling, in the sense that it may not be clear whether it reflects an adaptation of structure to potential functional differences or whether it simply reflects biological variability where any consequent functional differences do not play any important roles. The important issue thus becomes to what such morphological variability and diversity can be attributed? As recently pointed out in a detailed study of morphological variability among climbing fibers in the cerebellar cortex (Brown et al. 2012), if morphological diversity cannot be attributed to differences across anatomical regions, or to distinct subclasses within a more global class of neuron, it suggests that the variability is an irreducible population characteristic. In our study, where we recorded from and filled AII amacrine cells in retinal slices, we used the dendritic field size as a proxy measure of eccentricity and our analysis showed that the population of reconstructed AII amacrines most likely included cells from a wide range of eccentricities. At the same time, when we analyzed various morphological properties as a function of presumed retinal eccentricity, there was still considerable variability for a given retinal eccentricity. To our knowledge, the variability of different morphological properties, including dendritic field size, at a given retinal location has not been systematically investigated and is basically unknown. Whereas the difference in dendritic field size as a function of eccentricity has a direct functional counterpart in the size of the receptive field, and therefore reflects a functional adaptation, we do not know if the differences in neuronal morphology related to dendritic field size by themselves give rise to differences in functional properties, e.g. differences in dendritic integration and signal processing. Irrespective of whether we are primarily concerned with differences in morphology between or within different retinal eccentricities, such differences and their potential consequences for neuronal computation are receiving increasing attention. Given that dendritic morphology as such can have dramatic effects on neuronal function, it is easy to overlook the inherent variability in dendritic morphology between cells belonging to the same type of neuron and the consequences this might have for functional heterogeneity (Schneider et al. 2014).

In our material, we did not find evidence of clustering of morphological properties among AII amacrines and no evidence for the existence of subtypes of such cells. The idea of subtypes of AII amacrines might seem paradoxical, as it has been demonstrated repeatedly in different species that the population of AIIs is arranged in a regular mosaic (Vaney 1985; Mills and Massey 1991; Wässle et al. 1993, 1995; Casini et al. 1995), strongly suggesting that these cells constitute a unique and homogeneous population that subserves a specific function or set of functions in the retina (Sterling 1983). There were at least two reasons for investigating the potential heterogeneity of the population of cells we have reconstructed. First, because we did not target the cells on the basis of a population marker, there is no guarantee that all cells we filled and imaged would belong to the same population. We have indeed observed that with the criteria used for initial targeting (shape and location of cell body), we sometimes obtain different types of wide-field instead of AII amacrine cells (see Veruki et al. 2007 for examples), depending on the stringency with which the criteria are applied. The electrophysiological properties of wide-field amacrine cells are very different from those of AII amacrines, however, and fluorescent imaging has always revealed either wide-field-like or AII-like morphology. Rat retina contains other glycinergic, narrow-field amacrine cells (Menger et al. 1998), but with the criteria used for targeting AII amacrine cells, we have never encountered cells similar to those described by these authors. Second, the existence of subtypes of AII amacrine cells was recently postulated on the basis of differences in physiological response properties (Pang et al. 2012), but it is not clear to us that the differences reported by these authors reflect genuine clustering, as opposed to the presence of a continuum.

Apart from the expected difference in dendritic field size, reflecting a corresponding difference in receptive field size, it is not obvious how the morphology of individual AII amacrine cells would vary as a function of retinal eccentricity. Assuming that we have sampled AII cells across different eccentricities, we find it intriguing that the reconstructed population conforms to the scaling principle recently discovered by Teeter and Stevens (2011). When they investigated a large number of different types of neurons sampled from different brain regions, they found that branch density decreases with increasing arbor territory, with density defined as total length divided by arbor territory. This scaling principle held irrespective of whether different neurons occupied 2D (e.g. Purkinje cells and retinal ganglion cells) or 3D territories (e.g. cortical pyramidal cells). When we analyzed the reconstructed AII amacrines in the same way, they conformed to the same scaling principle. The parameters of the fitted function were slightly different, but can be easily explained by the lower range of values for branch density and territory volume covered by the AII cells compared to that of the cells analyzed by Teeter and Stevens (2011).

## Conclusions

The collection of morphologically reconstructed AII amacrine cells presented in this study may be the first detailed digital reconstruction of this cell type for any mammalian species. A database of quantitative data for a larger population of cells, as generated in this study, will provide a useful source of information that can be necessary for constraining larger-scale models of the rod pathway in the mammalian retina. In addition, it can serve as a reference for estimating the extent to which any individual cell is within the limits of “typical” morphologies and therefore the extent to which its properties are representative for the population as a whole. A database of quantitative morphological reconstructions can also be useful for investigations that address changes in cellular morphology evoked by pathological conditions, disease processes, and developmental mechanisms. Finally, the workflow established here for filling and imaging live neurons, followed by digital morphological reconstruction can be extended to encompass development of complete compartmental models of these neurons.
